# Development and validation of a health information system for assistance and research in gestational trophoblast disease

**DOI:** 10.1186/s12911-022-01916-4

**Published:** 2022-07-01

**Authors:** Jaqueline Martins, Paulo Bandiera-Paiva, Antonio Rodrigues Braga Neto, Lucas Ribeiro Borges de Carvalho, Lúcio Padrini-Andrade, Vitor Tonini Machado, Antônio Carlos da Silva Junior, Sue Yazaki Sun

**Affiliations:** 1 Department of Obstetrics, Universidade Federal de São Paulo, São Paulo, Brazil; 2 Department of Health Informatics, Universidade Federal de São Paulo, São Paulo, Brazil; 3 Department of Obstetrics, Universidade Federal do Rio de Janeiro, Rio de Janeiro, Brazil; 4 Neonatology Division, Universidade Federal de São Paulo, São Paulo, Brazil

**Keywords:** Gestational trophoblastic disease, Information science, Health Information System, Mobile applications, Computing metodologies, Telemedicine

## Abstract

**Background:**

Gestational Trophoblastic Disease (GTD) comprises pathological forms of placental trophoblastic tissue proliferation. When benign, they present with hydatidiform moles, and when malignant, they are called Gestational Trophoblastic Neoplasia. With the growth of the practice of digital health, allied to updated therapeutic approaches, the Outpatient Clinic for Gestational Trophoblastic Disease has built a Health Information System (HIS), contributing to the teaching–learning binomial, as well as to self-care.

**Methods:**

This is a cross-sectional and blind technological assessment research for developing SIS-Mola (Website for the medical team and the Application “MolaApp” aimed at patients with GTD). We used the Praxis management approach to manage the application creation project. In the tasks involving real-time chat, a WebSocket layer was created and hosted together with the project’s web services, which use the Arch Linux operating system. For the evaluations, we provided questionnaires developed based on the System Usability Scale (SUS), to determine the degree of user satisfaction, with objective questions on the Likert scale. We invited 28 participants for the evaluations, among ABDTG specialist physicians, doctors from the DTG Outpatient Clinic team, and the patients. The study was systematized according to the rules of treatment and follow-up in treating the disease.

**Results:**

The tests were conducted from November 2021 to February 2022. The responses obtained on a Likert scale indicated reliability and credibility to the HIS, since the total usability score, measured by the ten questions of the SUS instrument, had a mean of 81.1 (clinicians), 80 (patients) and median of 77.5 for both groups. The sample was characterized according to the variables: age, gender, education, computer knowledge, and profession.

**Conclusion:**

Developing a HIS in the GTD Outpatient Clinic met the objectives regarding the rules of treatment and follow-up of patients. With these digital tools, it is possible to obtain data about the patient’s health, sending information through exams performed and appropriate treatments. The connectivity capacity allows agile care, saving time, costs and solving the displacement problem. The TICs generate natural efficiency for the organization in the flow of service and the formation of a database, improving the quality of the assistance.

**Supplementary Information:**

The online version contains supplementary material available at 10.1186/s12911-022-01916-4.

## Background

Gestational Trophoblastic Disease (GTD) comprises pathological forms of placental trophoblastic tissue proliferation. When benign, it presents the complete hydatidiform mole (CHM) and partial hydatidiform mole (PMH). When malignant, it shows the invasive mole, the choriocarcinoma, a trophoblastic tumor of the placental site, and the epithelioid trophoblastic tumor, Gestational trophoblastic neoplasia (GTN) [1, 2]. According to the International Federation of Gynecology and Obstetrics (FIGO) [3], it is recommended that specialists follow the patient during the initial diagnosis and the post-molar follow-up to monitor the dosage of the regression curve of the Human chorionic gonadotropin (hCG) hormone, her hormonal biomarker. It is necessary to diagnose early progression to GTN, with the initiation of chemotherapy performed by chemotherapy in most cases. Thus, with adequate treatment, there is a chance of a high cure rate and the possibility of a healthy pregnancy [4]. From this perspective, through correct decisions, diagnostic and therapeutic actions for disease prevention and recovery are based on research, exchange of information, and scientific evidence.

In this sense, with the growth of the practice for digital health and the need for clinical follow-up combined with updated therapeutic approaches, the Gestational Trophoblastic Disease Outpatient Clinic, one of the Reference Centers in Brazil, by the University Hospital/Hospital São Paulo, Escola Paulista de Medicina, from the Federal University of São Paulo (HU/HSP/EPM-Unifesp), specialized in the treatment, care, and research, undertook the construction of a Health Information System (HIS), composed of two simultaneous interfaces: a Website (responsive site) for the medical team, which, associated with an Application (MolaApp) aimed at patients, contribute to the teaching–learning binomial, as well as to self-care [5].

With regard to inserting Information and Communication Technologies (ICTs) in the provision of health services, it impacts changes in the professionals' learning process by establishing a new “doctor-internet-patient” relationship, considering quality, safety, security, and efficiency in the assistance provided [6].

From this real-time or remote computing, these tools can help treatments in different geographic regions, reduce time and cost and improve communication quality, promoting specialized coverage of health care that can be disseminated to other GTD Reference Centers in Brazil.

Another essential factor for the educational assistance of these patients undergoing GTD treatment, their families, and other interested people is the use of social media and digital platforms that enable pedagogical and innovative initiatives for health education. Diniz et al. [7] report the experience of online interaction, through a Facebook page, between the directors of the Brazilian Association of Gestational Trophoblastic Disease (ABDTG) and patients with hydatidiform mole, whose study showed positive results in terms of satisfaction, security and understanding of the information and guidance provided, even remotely.

In a recent study, results obtained in a data survey regarding self-care and benefits promoted to the psychological, social, and financial aspects of the patients emphasize the importance of telemedicine for post-molar care and follow-up in the comparison between being a face-to-face care or through a mobile devices, via WhatsApp [8]. This study was carried out in the same Reference Center in GTD of our research.

In addition, in similar scenarios, Paulista Associtation of Medicine (APM) surveyed 1,614 professionals to map the use of digital solutions in routine care in the state of São Paulo (Brazil), where 82.65% of physicians use the technology in their day-to-day, to observe patients or to optimize appointments. The objective of this study was to map the use of technological resources in Medicine and Health, and 67.66% of respondents agreed with the phrase “Technology will not replace the doctor, but it can replace the doctor who does not use technology”. And, in fact, among the interviewees, 93.68% understand that sharing information benefits everyone involved [9].

Other literary studies have analyzed the use of telemedicine to facilitate the provision of services associated with health care, including cases in which distance is a critical factor. They showed that research related to the use of digital tools attested to a good gain for the relationship between health professionals and their patients, with the insertion of the “Meu Pré-Natal” Application (women during pregnancy, childbirth, and the puerperium) [10].

In the same way, the results obtained in the “Evaluation of Mobile Applications for Health Promotion of Pregnant Women with Pre-Eclampsia” proved the possibility of verifying the reliability of the tools, as well as the information being consistent with promoting health and the quality of life of these pregnant women [11].

The motivation for the HIS construction was due to the lack of published works that portray the interaction between two technological devices developed, with a simultaneous application, as tools to help in the process of treatment of GTD, and by the diffusion of frequent access to computers, tablets, and smartphones, as a facilitator in communication.

For improved care and distance services to represent a significant opportunity in needy and remote regions, where specialized face-to-face care is not available, the application of low-cost technologies is necessary. It should be encouraged the implementation of integrated collaborative networks for remote assistance [12].

In this sense, with the advancement of ICTs, mobile applications aimed at the health area act as tools for self-management in diseases aiming at the process of mutual responsibility, which facilitates the achievement of clinical results in the conduct of treatments and a promising proposal for the self-care at home [12].

Nowadays, cell phones are no longer used only for phone calls or receiving and sending messages. They are increasingly developed with advanced technologies and data storage capacity for the benefit of users [13].

When it comes to ethical and legal aspects, it can be highlighted that technological advances are impacting all health network processes, from communication to information collection. Its merger affects all dealings related to new medical procedures and other health procedures. In Brazil, norms and regulations defined therapeutic guidelines that subsidized the insertion of ICTs, due to the changes that occurred during the Covid-19 pandemic [14–16].

Given these considerations, the question that guided the development of this research was: “What are the advantages and benefits of developing a system capable of promoting adequate post-molar follow-up and early diagnosis of its malignancy called Gestational Trophoblastic Neoplasia through ICTs?”.

## Methodology

This is a transversal and blind technological evaluation research for the development of a Health Information System (HIS), composed of two interfaces: The website (responsive site) for the activities of the medical team and the Application (MolaApp) for the patients. The project phases were defined by techniques used by software engineering, which included requirements analysis, programming, and development of digital tools, as well as application of tests and evaluation questionnaires. The study was developed according to the rules of care and follow-up at the GTD Outpatient Clinic—HU/HSP/EPM/UNIFESP.

For this undertaking, a team was formed with a project manager, a requirements analyst, two programmers, and four systems analysts. The requirements analyst belongs to the Discipline of Neonatology, and the others belong to the Project Office of the Department of Health Informatics at Unifesp. In addition, we had the assistance and supervision of the advisor and coordinator of the GTD Ambulatory, as well as the Technical Assistance in Health Informatics's co-orientation of this work.

Bibliographic surveys and literature review were performed in databases indexed in PubMed, Scielo, Lilacs, and Web of Science.This research was submitted and approved by the Research Ethics Committee of the Federal University of São Paulo, on March 27, 2020.

### Project management method for the development of the Health Information System

For the project management of creating the application, website, and mobile application, we used the Praxis [17] project management approach. This approach was selected because it is free to use, flexible to our needs, and comprehensive. It offers a body of knowledge, methodology, competency framework, maturity model, and terminology in a single integrated structure.

**Fig. 1:**
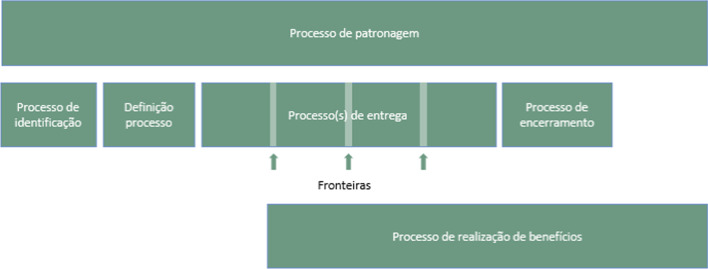
Praxis Framework project and program process [17]. *Source*: https://www.praxisframework.org/en/method/project-and-programme-processes

The approach involves the components presented (Fig. 1) and described below:

- Patronage process: According to the Praxis Framework, it is the process that “a patron must undertake to exercise overall control and make key decisions during the life cycle”. This process was assisted by the project manager and conducted by the coordinator of the GTD Ambulatory and supervisor of this research, ensuring the medical team’s perspective and requirements. It was part of these steps: meetings at the end of each downstream process and during the handover process for scope adjustment and follow/no follow decision making.
- Participating team: Specialist GTD coordinator and project manager.
- Identification process: The initial stage—During this process, a project summary was developed, the effort necessary to define it in-depth, and submitted to the authorization of the project sponsor.
- Participating team: Specialist GTD coordinator, one project manager, one requirements analyst, two programmers, one system analyst.
- Definition process: In this step, the project plan is fully developed. A task list with reliable estimates, an arrow diagram that later became a gantt chart, an entity-relationship graph, and a description of the project's governance guidelines were created.
- Participating team: Specialist GTD coordinator, one project manager, one requirements analyst, two programmers, and one system analyst.
- Delivery Process: This is the process in which the Website and MolaApp application were developed. It has been divided into development cycles with go/no-follow decision-making boundaries. It allows scope changes and accommodates dependencies and risks encountered during the delivery process that were not anticipated in previous methods.
- Participating team: 1 project manager, one requirements analyst, two programmers, and one system analyst.
- Closing Process: Tasks performed are compiled, reviewed, and accepted by the project sponsor. Products (MolaApp website and app) are delivered, and lessons learned during all processes are reviewed and discussed.
- Participating team: Specialist GTD coordinator, one project manager, one requirements analyst, two programmers, and one system analyst.
- Benefits realization process: Developing products does not automatically bring benefits. In this research, this process comprised the Validation and evaluation of this Health Information System.
- Participating team includes nine doctors from the GTD outpatient clinic—HU-HSP/EPM/UNIFESP, five from the ABDTG, and 14 patients with GTD.

**Fig. 2:**
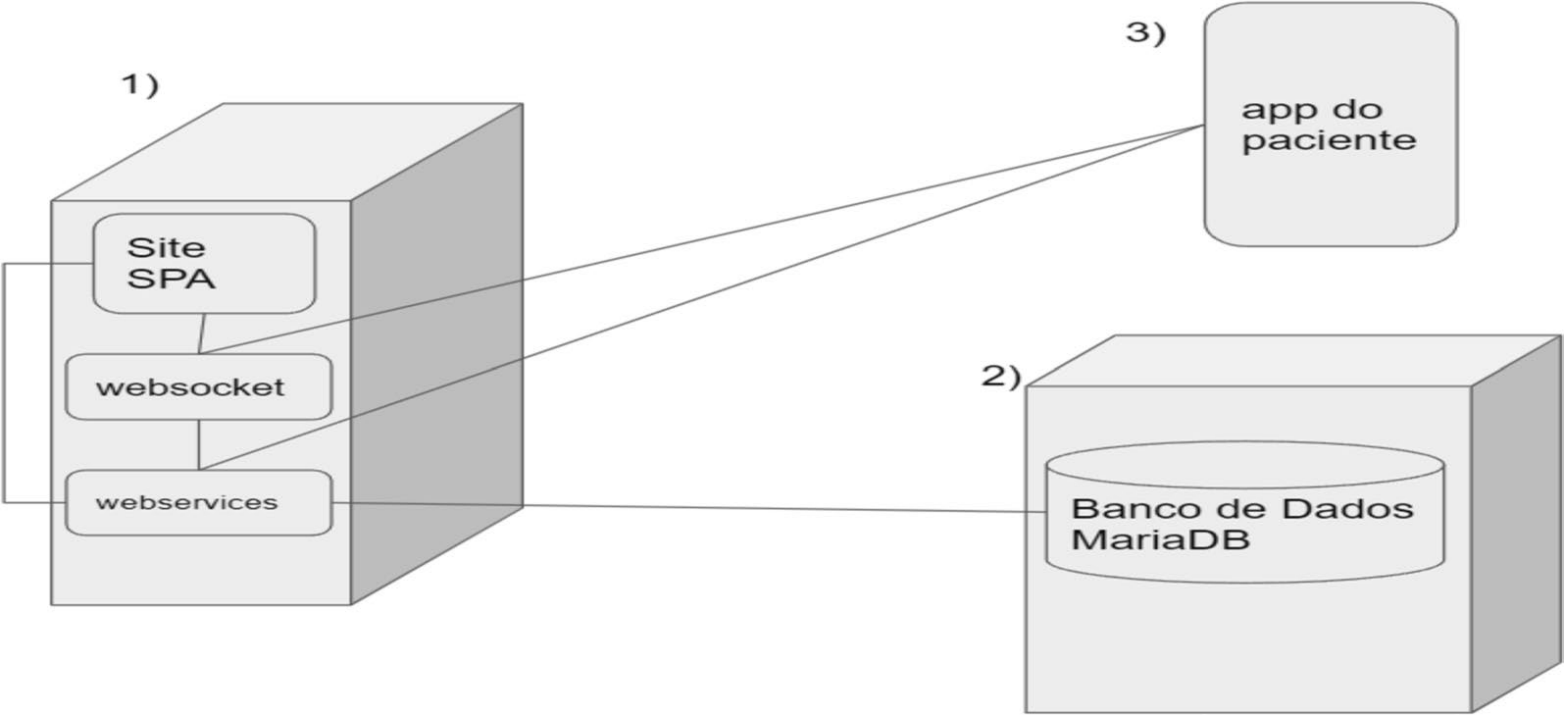
Application services architecture covering server 1, containing web services, websockets, single page application (SPA) site, which is the medical team’s interface; server 2 with a MariaDB database; and the android application, which is the patient interface [19]

Consequently, in the delivery process, the development tasks were performed using a services-oriented architecture [18] comprising two interfaces (medical staff and patients) and intermediated by a services layer that communicates between the interfaces and the data bank. For tasks involving real-time chat, a WebSocket layer was created, and hosted with the project's web services. This project was hosted on two servers and used an application interface made for patients, as shown in Fig. 2.

The servers use the Arch Linux [19] operating system and are divided as follows:

1. A database server using the MariaDB [20] database management system to store application data and maintain consistency between parts of the system.
2. A server with web services made with nodeJS [21] that maintains the services used by the website and the mobile application and communicates with the database. The SPA site is also hosted on this same server, using the Quasar Framework [22]. A WebSocket was also hosted on this server using socket.io [23] to mediate real-time chat communication between the physicians' and patients' interface using the vue-socket [24] library.
3. An application with patient interfaces developed using the Quasar framework [22] and exported using the integrated cordova library.

### HIS: web interface and MolaApp application

The Health Information System about Gestational Trophoblastic Disease (GTD) and related Neoplasms was intended to provide patients and their families with agility in sending the results of collected exams, obtaining information, and providing clarifications in case of need via the internet. line. Providing the medical team with the opportunity to record longitudinal clinical data from the treatment.

### System scope and users

**Fig. 3:**
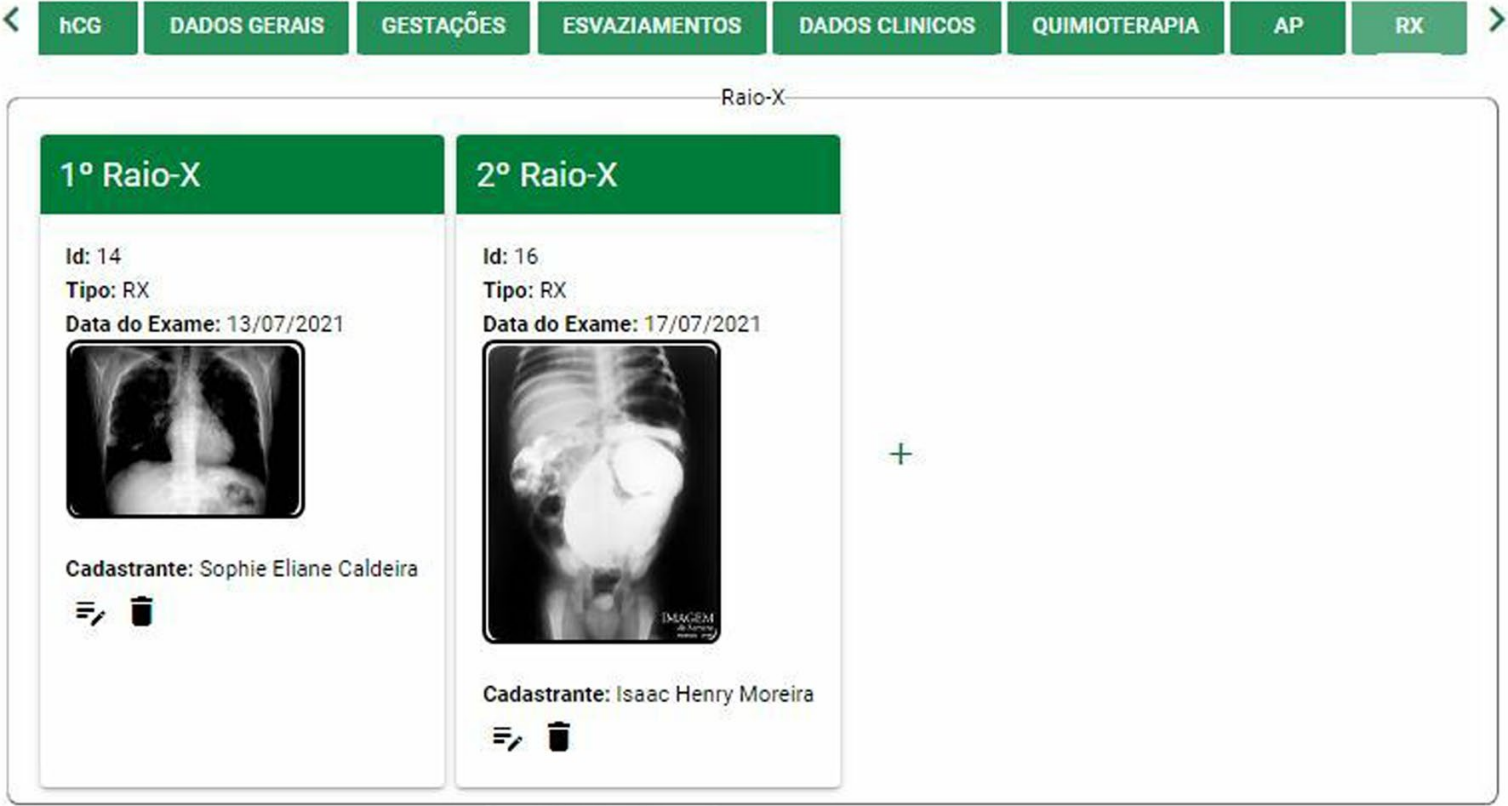
Submission of exams: X-ray screen

- Web Interface: The Website contains all resources for registering patients with their personal, clinical, and demographic data, encompassing all activities relevant to the GTD Ambulatory. For example, below we have the screen for organizing the x-rays sent by patients (Fig. 3)

The coordinator has the attributes of all registered profiles of the medical team (professor, preceptor, residents, and interns), in addition to managing all HIS functionalities, via the Website or Application. You will be able to issue individual reports of patients or a group of patients, either longitudinally, to view the treatment, or transversal, to observe only one event, via the Web platform.

The doctor performs the consultations and will include the clinical data and updates of the registration data through the Web environment and will be able to view the report of these and send messages and guidelines via chat.

**Fig. 4:**
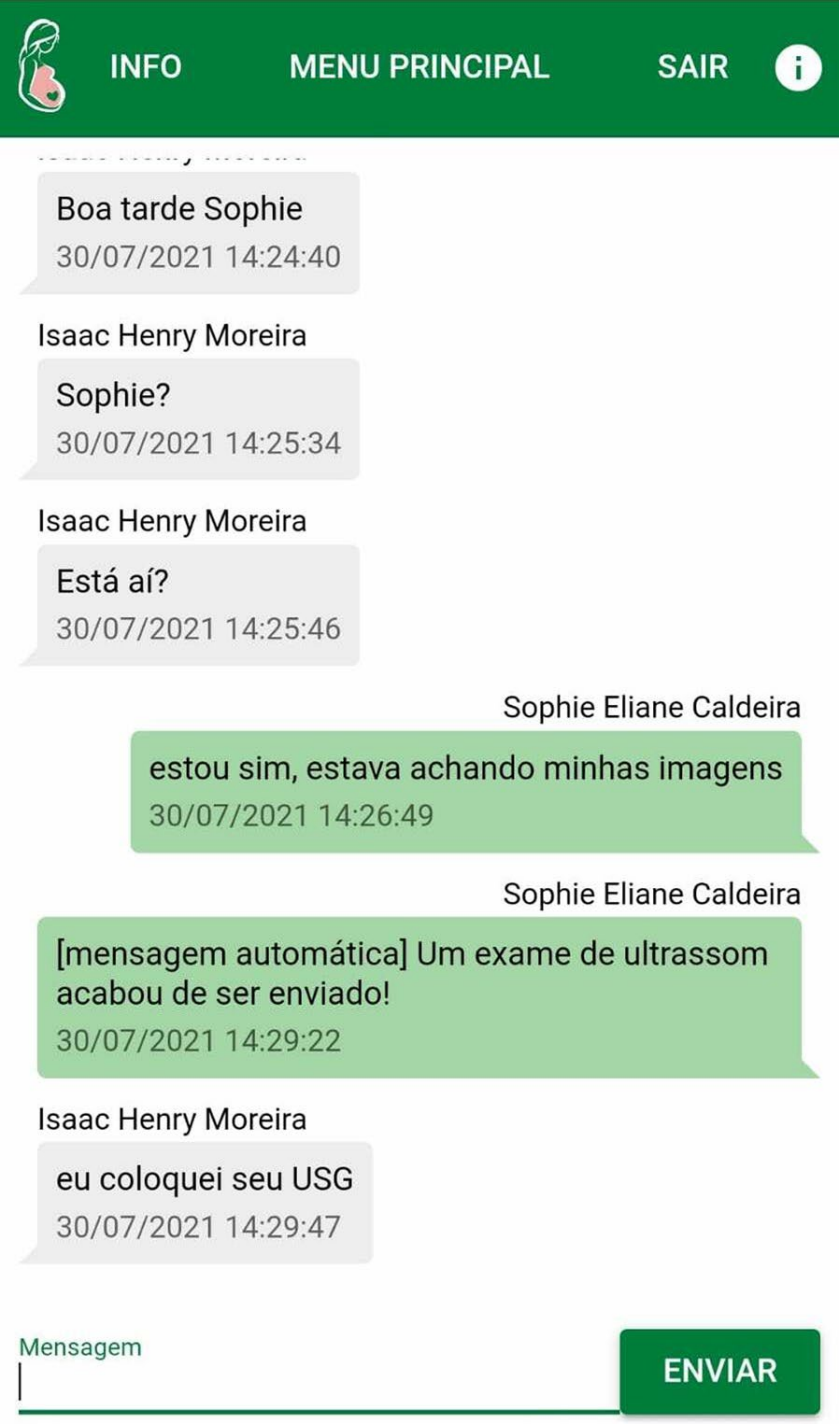
Chat’s screen between physician and patient (MolaApp)

- MolaApp Application: It will be used exclusively by the patients in attendance to assist in the general communication, interaction, and follow-up of the treatment (Fig. 4).

When performing tests in other laboratories, patients may include clinical data, accompanied by reports or photographs, via the MolaApp Application. They will have a longitudinal follow-up of the results obtained by the doctor who performed the care. In addition, they will receive alert messages regarding the frequency of exams or when there is a variation from one exam to another so that they can contact the Reference Center.

Both the Website and the MolaApp Application developed were composed of concepts, risk factors, signs, symptoms, treatments, importance of medication and side effects, frequent questions, necessary health care and patient follow-up through monitoring, with the purpose of provide clarification, encourage therapeutic care and the importance of continuity in treatment. The User's Manual (in Portuguese) is provided as Additional file 1.

### Evaluation of quality of use and functionality

Five specialists in GTD, who are members of the ABDTG study group, were invited to coordinate the care provided in Brazil [25], as well as nine doctors from the GTD Ambulatory team (HUHSP/EPM/UNIFESP) and 14 patients with the diagnosis. After accepting the Terms of Free and Informed Consent, a brief personal training took place to clarify the actions to be carried out with the devices.

Regarding the evaluations, we provide questionnaires developed in a REDCap environment, followed by an Instruction Manual for the use of the Mola System, containing illustrative images that help understand the step-by-step process for carrying out practical activities (complementary material).

The Questionnaire of Patients with Gestational Trophoblastic Disease (https://redcap.epm.br/surveys/?s=RTCKCLRKP4MLXETF) is composed of two parts. The first was for demographic questions of the participants, and the second with ten questions from the System Usability Scale and an optional essay question [26].

**Fig. 5:**
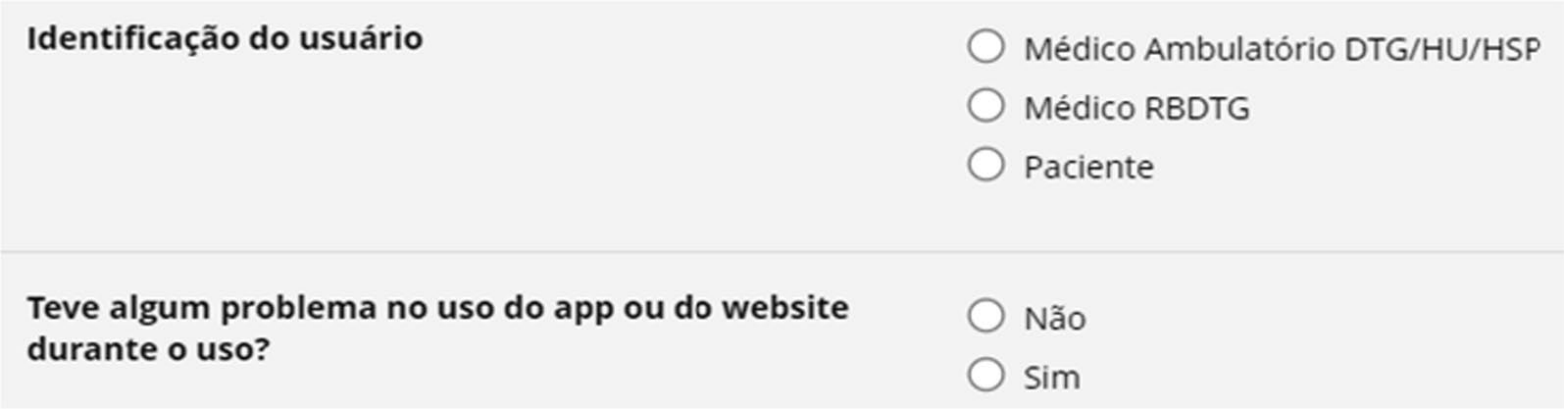
Occurrence questionnaire

The Occurrence Questionnaire (https://redcap.epm.br/surveys/?s=RLKMXP8ANEKEPDEE) identified the user, doctors, or patients and recording problems in the use of the website or the MolaApp application (Fig. 5).

## Results

The tests were carried out from November 2021 to February 2022, and 28 eligible participants (14 in both groups) were invited to carry out the usability and functionality assessments for the study, distributed among physicians from ABDTG, physicians from the DTG outpatient clinic, and patients undergoing treatment. Fourteen responses were obtained, 9 clinicians and 5 patients, which represents a 50% return rate.

### Sample characteristics

The clinicians and patients’ sample was characterized in relation to the variables: age, sex, education, computer knowledge and profession (Tables 1, 2).

**Table 1:** Clinicians’ sample characteristics (n = 9)

Characteristics	
*Sex*	
Female	5 (55.6)
*Age*	
Mean (standard deviation)	45 (15)
Median	43
Minimum–maximum	29–78
*Education*	
Graduation	1 (11.1)
Specialization	4 (44.4)
Master degree	0
Doctorate degree	4 (44.4)
*Computer knowledge*	
Basic	3 (33.3)
Intermediary	5 (55.6)
Advanced	1 (11.1)

**Table 2:** Patients’ sample characteristics (n = 5)

Characteristics	
*Sex*	
Female	5 (100)
*Age*	
Mean (standard deviation)	34 (8)
Median	32
Minimum–maximum	27–47
*Education*	
Graduation	3 (60)
Specialization	2 (40)
*Computer knowledge*	
Basic	2 (40)
Intermediary	3 (60)

### User’s satisfaction evaluation

**Fig. 6:**
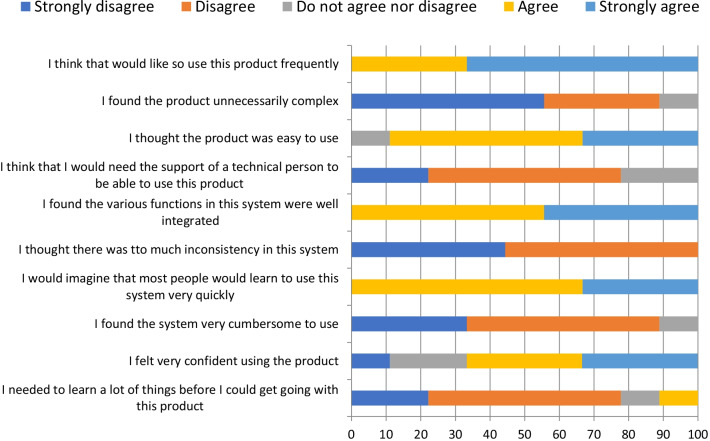
System usability scale: clinicians’ evaluation (%)

**Fig. 7:**
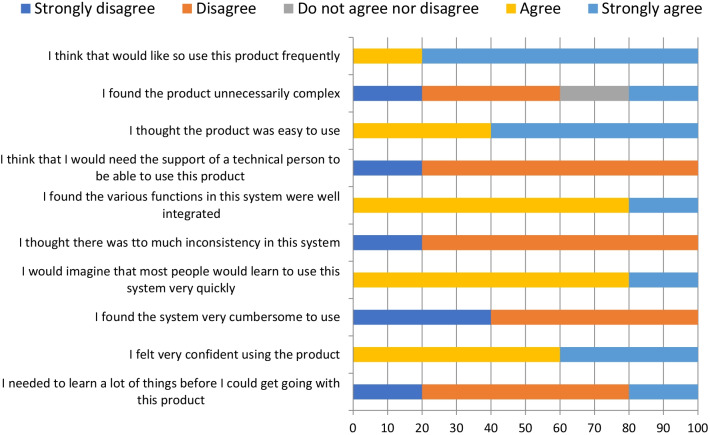
System usability scale: patients’ evaluation (%)

After the evaluation phases, using the System Usability Scale—SUS, to determine the degree of user satisfaction with the final product, website by clinicians, and app by patients, the questionnaire contained ten objective questions graded on a Likert scale, with values from one to five, classified respectively such as: “strongly disagree”, “disagree”, “do not agree nor disagree”, “agree” and “strongly agree” (Figs. 6, 7), and an optional essay question. Data were collected in a form developed in REDCap and analyzed in Microsoft Excel 2010 for Windows® and R software, version 4.1.2. [26].

**Fig. 8:**
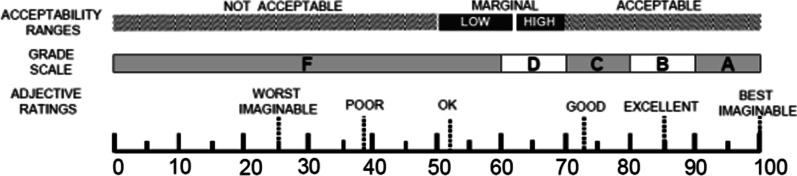
SUS classified scale

The total usability score measured by the ten questions of the SUS instrument presented a mean of 81.1 for the clinicians’ group and 80 for the patients’ group. The median was 77.5 points for both groups. All results were classified by excellent (72.5–85), considering adjective scale and acceptability ranges (> 68), showed in Fig. 8 [27].

Accurate quality of use is related to the ability and ease of users to achieve their goals with efficiency and satisfaction, communicability (quality of doctor-patient communication through the interface), and applicability (usefulness in different situations) [28].

About the questionnaire of occurrences during the use of the system, 13 responses were obtained, where 11 indicated the absence of problems, and two doctors from the HU-HSP reported having difficulty accessing the system.

### Association analysis

The association between age and SUS Score was studied using Pearson's correlation coefficient since the SUS Score variable showed adherence to the normal distribution by the Shapiro–Wilks test (*p* < 0.05). The Pearson correlation result for clinician’s group was − 0.541, which means a low correlation, and − 0.110 for the patients' group. In this group, the correlations were significantly and inversely proportional; the younger the age, the higher the SUS score.

## Discussion

Even though only 50% of the total number of invited participants, the amount was enough for the users to validate the system.

According to studies already carried out to measure the minimum number of participants in evaluations, five users are needed among the categories per performance. In the case of different groups, such as the developed HIS (doctors and patients), five users are also recommended. Therefore, with this selected group, it was possible to cover up to 80% of usability problems that could have been found [29].

On the other hand, too, research suggests that there is no consensus on the ideal size. However, five users may underestimate the number of errors, concluding that they should use eight to nine users to eliminate problems with a small sample size [30].

The present study had 14 users, representing a sample above the minimum numbers indicated for usability tests and their validation.

All participants assessed usability through a questionnaire based on the System Usability Scale (SUS) during the tests. The answers to the questionnaire showed good reliability and credibility to the developed system. The SUS score for physicians and patients was 81.1 and 80, respectively, is classified as excellent and acceptable according to the SUS Adjective Rating Scale [27].

The association of age with the SUS score by Pearson's correlation was − 0.541 (physicians) and − 0.110 (patients). The negative number represents an inversely proportional relationship between the two variables, and its correlation varies between − 1 and + 1; that is, the value should be close to the extremities, which occurred with the patient group. The group of participants regarding academic training presented similar results.

Regarding usability, being the individual's ability to understand and operate a software used for specific purposes [31], in the feedback from the evaluations, this step was confirmed by the participants.

According to the literature, a SUS score above 68 indicates an acceptable degree of usability [32]. An extensive analysis of the SUS questionnaire’s application identified that a score of 85 would be associated with excellent acceptance of a software or an application [27]. In this sense, the average SUS score for evaluating the system's usability reached the parameters described in the literature [27].

The choice of SUS for application in health was based on the number of questions, reliability, interpretation of the score, ease of deployment and online distribution, and previous studies carried out in the health area [33–37].

The System Usability Scale (SUS) is a research instrument, developed by Brooke in 1986, which contains ten questions that aim to measure the usability of various products and services. In relation to other existing assessment instruments (ASQ, CSUQ, PSSUQ, USE, SUMI, WAMMI), it has many advantages. First, the instrument is technologically agnostic, so it can be used to evaluate various products and services, including websites, hardware, multimodal systems, voice command systems, mobile applications, and clinical systems [33, 36].

This instrument is highly robust and versatile for usability professionals. In addition to the features mentioned above, the survey becomes relatively quick and easy to be used by both participants and administrators. Finally, the instrument generates a single score on a scale that is easily understood by most of those involved in the project, from project managers to programmers. This point is important, as the actors involved may have little or no experience in human factors and usability. One of the biggest advantages of the System Usability Scale is that there is no copyright, making the cost recommendable [34].

It is also easy to administer, has good reliability and references that help in the interpretation of its score [36].

The present study is a pioneer in this path for the benefit of patients and public health, as it presents the first description of a Health Information System with two simultaneous interfaces—Website and Application—for Brazilian mobile devices, developed for.

Furthermore, in the results obtained by the SUS, they reported that they were able to carry out all phases of the product interfaces, which configures capacity and ease of handling, as well as quality and satisfaction. In this definition, the concepts of usability are inserted [38]. Also, it was found that in the feedback from the occurrence questionnaire, among the 14 participants, 11 indicated that they did not find any difficulty with the SIS, two doctors had problems accessing but managed to participate, and one person did not report any occurrence.

When it comes to most apps, the most popular is for exercise tracking, diet tracking, and weight management. Some still help monitor blood pressure, blood glucose, pregnancy, menstrual cycle, and medication [39].

For the development of the Health Information System, we understand the importance of composting it with requirements that include several factors and necessary care with the health of GTD patients, through monitoring, to provide clarification, encourage therapeutic care, continuity of treatment, the promotion of self-care and adherence to digital tools.

In this sense, the HIS incorporates a routine in patient care and specific activities and speeds up data entry and notifications through monthly, biweekly, or weekly self-monitoring. The System was also developed based on the best available scientific evidence, clinical experience, and the expertise of nationally and internationally renowned GTD specialists [3, 8].

Outcomes in HIS presentations, in person or via web conferencing, compared to assessments and tests that participants performed and experienced individually, intensified the effects of expected results. At this point, the need to expand the role of health professionals and patients in self-care education [40, 41] should be recognized to make conducts that are relevant for the treatment, as well as economically, accessible to the target audience. viable, mainly for providing safe and free health practices, reaching a large number of low-income people and, sometimes, with difficulties in accessing Health Units. In addition to enhancing the exchange of information, reducing time and cost, and improving the quality of communication between doctors and patients.

As for the HIS interfaces, in general, they were considered by the participants who evaluated them as clear, adequate, accessible, practical, and satisfactory in the interactions, as well as the Instruction Manual made available to clarify doubts about the use of the tools that helped in the step by step for activities. However, it is essential to clarify that, although digital tools can help and support a medical team in terms of agility to obtain results, clarify doubts, request new exams or control appointments, this doctor-patient relationship will not exclude the face-to-face act, when necessary.

It is worth remembering that, after the advent of the Covid-19 pandemic, technology has proved indispensable in people's routines, as it is impossible to think about the quality of life without considering its contribution to health. The advances are numerous, with efficient, safe operating systems and modern equipment. The adoption of electronic medical records and advances in software engineering allow, through mobile devices, the performance of professionals with technological support that allows greater effectiveness in treatments and diagnoses [42, 43].

The study carried out at the same GTD Reference Center emphasized the importance of telemedicine for post-molar follow-up, through WhatsApp, through the data collected led us to think about officializing the organization of a database for all activities of the GTD Outpatient Clinic in the treatment of this disease and for the benefit of patients [8, 44].

## Limitations

Finally, one of the study's limitations was the lack of time and resources so that the MolaApp Application, at this moment, could also operate on the iOS system, which will be adequate in the future and is in the programming phase, in continuity to this research. And in the same sense, insert the suggestions given by the participants during the presentation and evaluation of the HIS, such as: “Does not recommend access to interns”; “Insert icons with text from other exams”; “Insert in the clinical data of a patient who does not menstruate and create a justification field”; “Insert a field in the clinical data to report other observations”; “Menstrual Calendar: insert contraceptive method, “which indication and relate to table” and in “chemotherapies cards: insert field to record all drugs used, for the improvement of the developed system”.

## Conclusion

The development of a Health Information System at the GTD Ambulatory, one of the Brazilian Reference Centers for the treatment of the disease, showed in its final results to meet the desired objectives, regarding the rules of treatment and follow-up of patients, since GTD it can present significant clinical complications, being very important the post-molar follow-up, for the monitoring of the hormonal dosage of hCG, thus preserving the patient's life.

In this way, the advantages of using these digital tools were obtaining data on the patient's health, sending accurate information through tests performed, and helping the medical team in the detection and possible pertinent treatments. Another key point is related to the connectivity’s ability to allow quick service, appointment scheduling, and exam requests, which saves time, costs, and travel difficulties. In addition, ICTs generate a natural efficiency for the organization in the flow of service and the formation of a database, allowing accurate studies for continued education and professional and scientific improvement in health to improve the quality of care provided.

## Supplementary Information

**Additional file 1.** System and User's Manual.

## Data Availability

The data used and analysed during the current study are not publicly available due Federal University of Sao Paulo policy but are available from the corresponding author on reasonable request.

## Abbreviations

GTD: Gestational trophoblastic disease
ABDTG: Brazilian Association of Gestational Trophoblastic Disease
SUS: Sytem usability scale
CMH: Complete hydatidiform mole
PHM: Partial hydatidiform mole
GTN: Gestational trophoblastic neoplasm
FIGO: International Federation of Gynecology and Obstetrics
hCG: Human chorionic gonadotropin hormone
HU: University Hospital
EPM: Paulista School of Medicine
UNIFESP: Federal University of Sao Paulo
HIS: Health Information System
ICT: Information and Communication Technologies
APM: Paulista Association of Medicine
