# Supplementary material for: Development and validation of a health information system for assistance and research in gestational trophoblast disease
Source: BMC Med Inform Decis Mak. 2022 Jul 1;22:173. doi: 10.1186/s12911-022-01916-4 (PMC9247895; doi:10.1186/s12911-022-01916-4)
Supplement: Supplementary file 1 — Additional file 1. System and User's Manual. [file 12911_2022_1916_MOESM1_ESM.pdf]

UNIFESP

UNIVERSIDADE FEDERAL DE SÃO PAULO

1933

INSTRUÇÕES PARA UTILIZAÇÃO DO SISTEMA MOLA (WEB e APP)

Universidade Federal de São Paulo
Hospital Universitário / Hospital São Paulo
Ambulatório de Doença Trofoblástica Gestacional

2021

UNIFESP

UNIVERSIDADE FEDERAL DE SÃO PAULO

1933

MÉDICO

Universidade Federal de São Paulo
Hospital Universitário / Hospital São Paulo
Ambulatório de Doença Trofoblástica Gestacional

2021

MÉDICO

- O endereço da plataforma web é https://dtg.bioinfo.unifesp.br

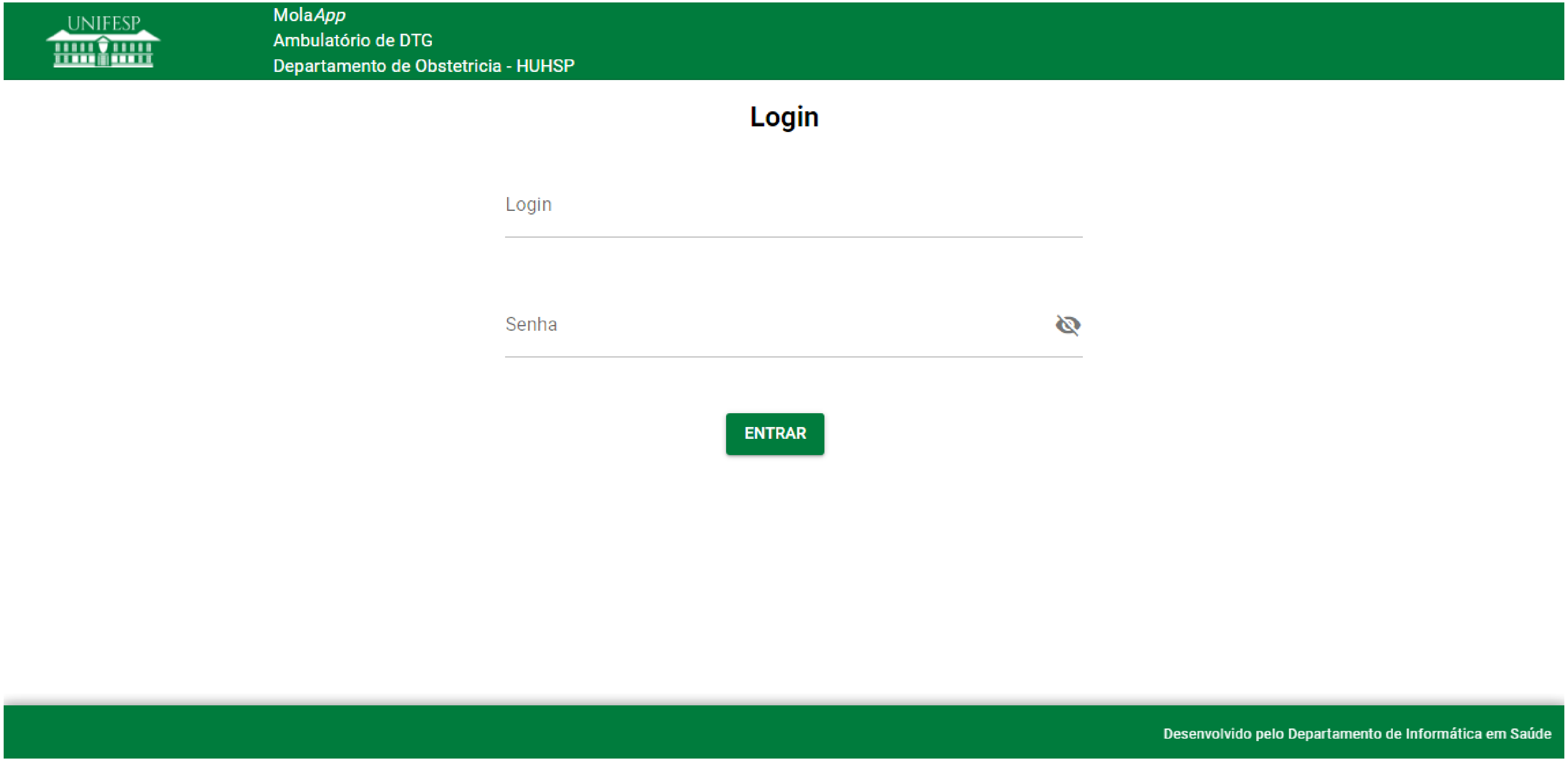

MÉDICO

- **Digitar login cadastrado no sistema pelo coordenador**
- **No primeiro acesso, a senha será igual ao login. A senha deverá ser alterada pelo usuário.**

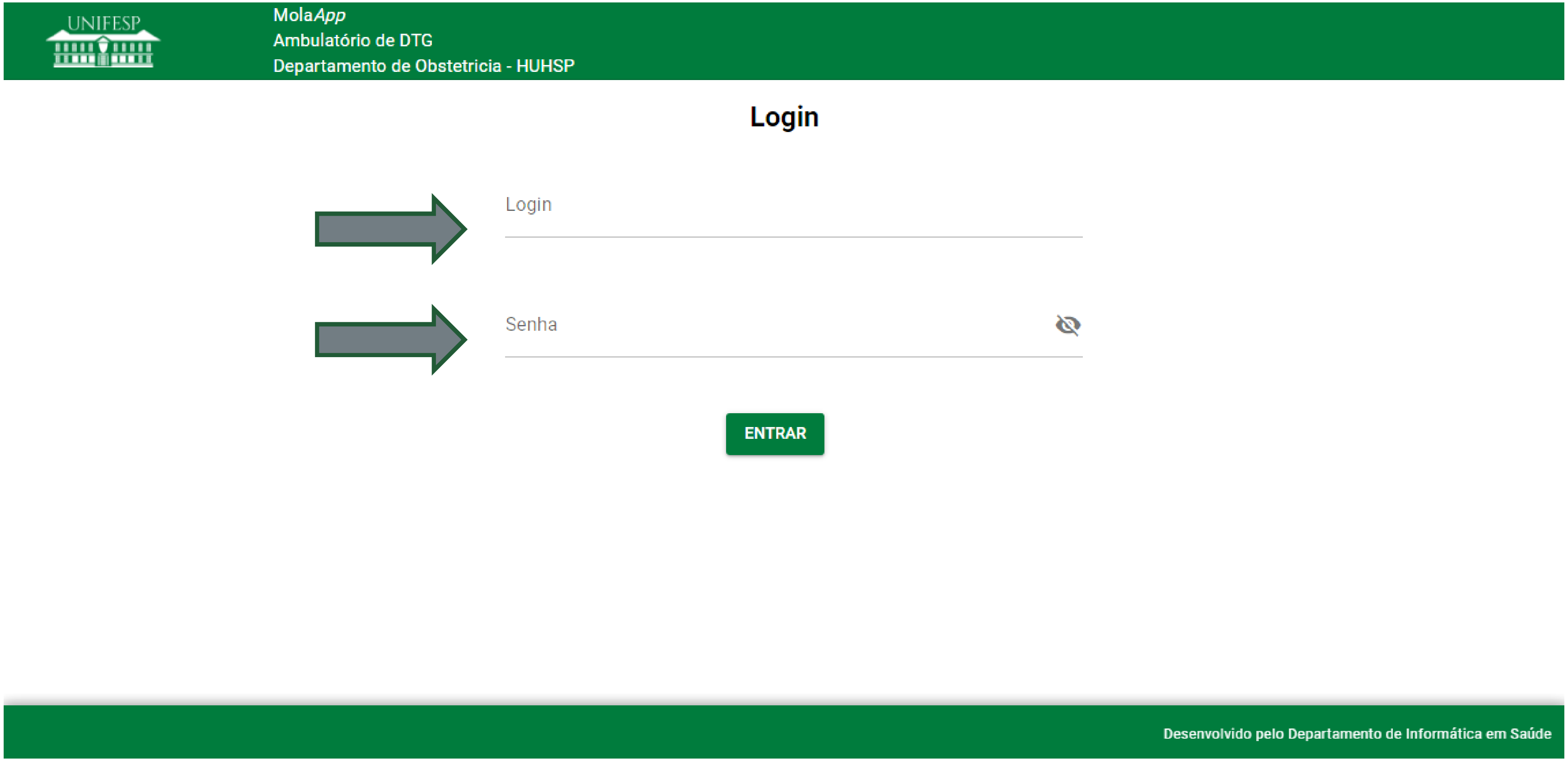

MÉDICO

ÁREAS

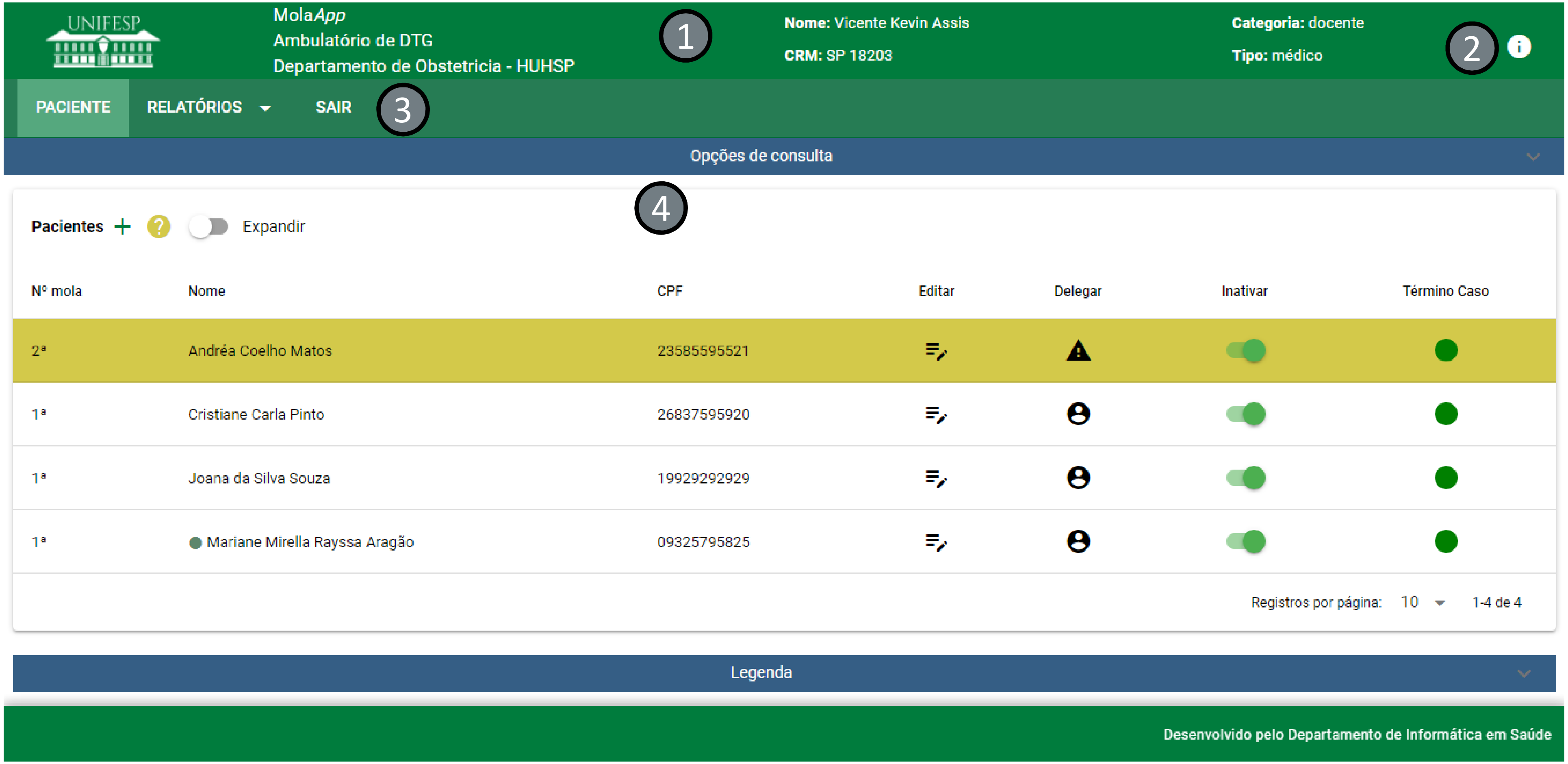

1 – Identificação do usuário
2 – Informações e troca de senha
3 – Seleção das telas para paciente e relatórios
4 – Visualização das seleções de paciente e relatórios

MÉDICO

INFORMAÇÕES

- **Nesta página encontra-se informações sobre o projeto de pesquisa, troca de senha do usuário e termo de aceite com aspectos legais sobre telessaúde.**

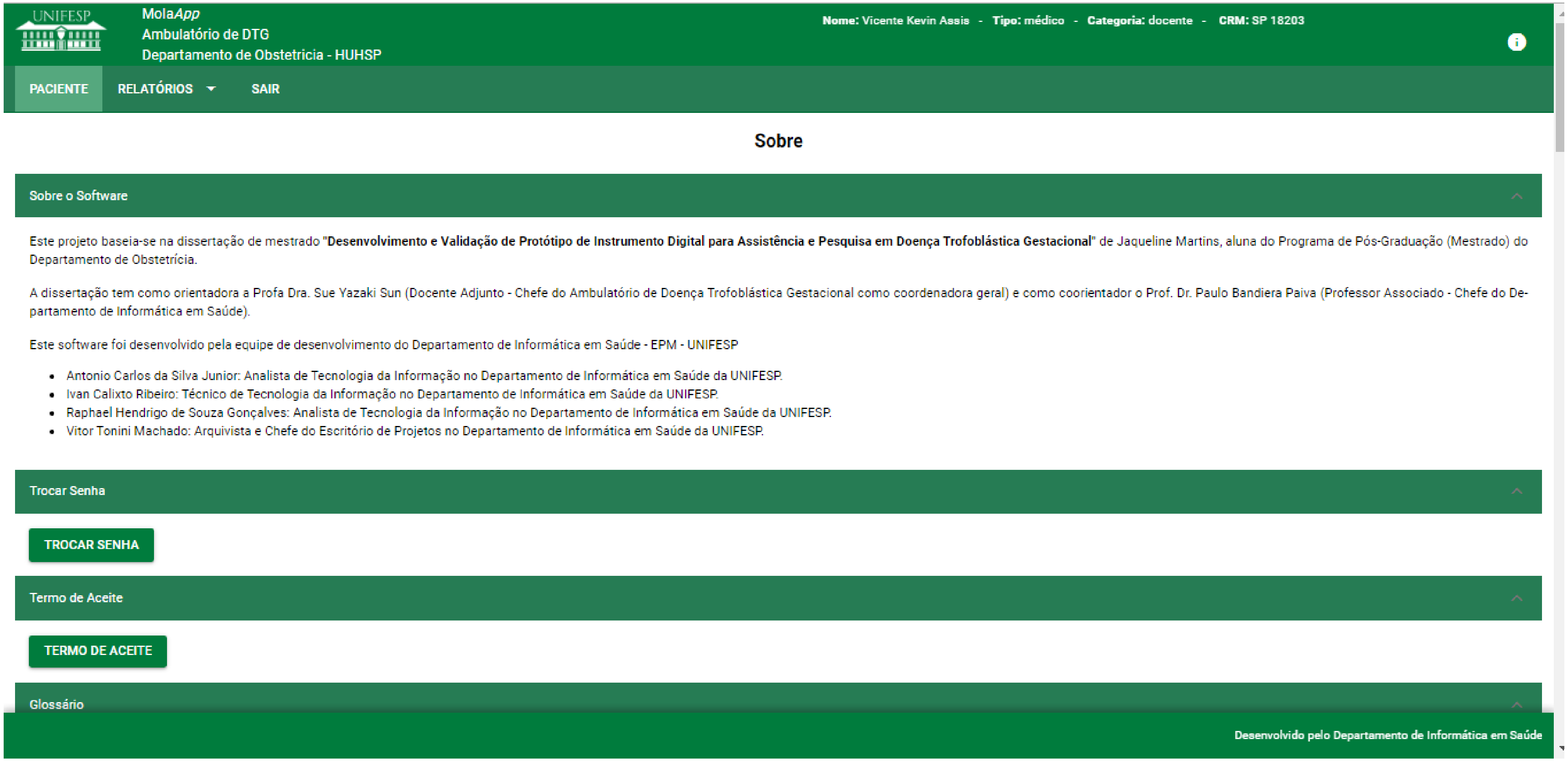

MÉDICO

PACIENTE

- **Nesta página encontra-se informações sobre os pacientes que estão sob os cuidados do usuário médico**

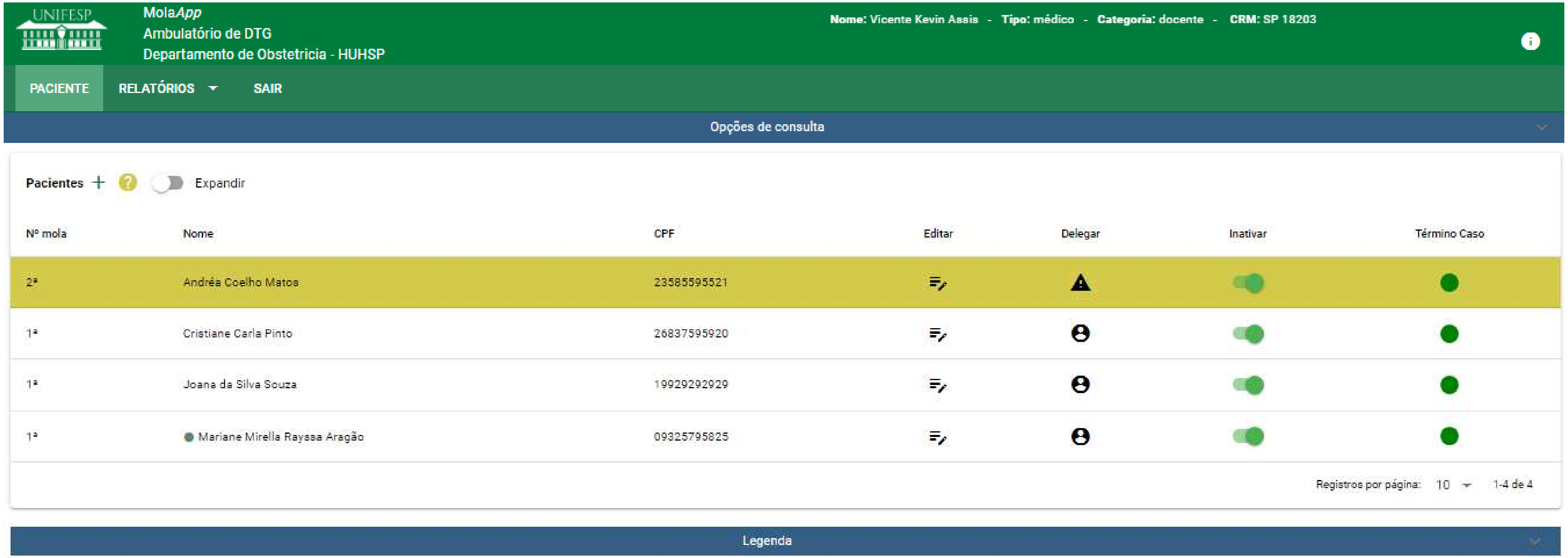

MÉDICO

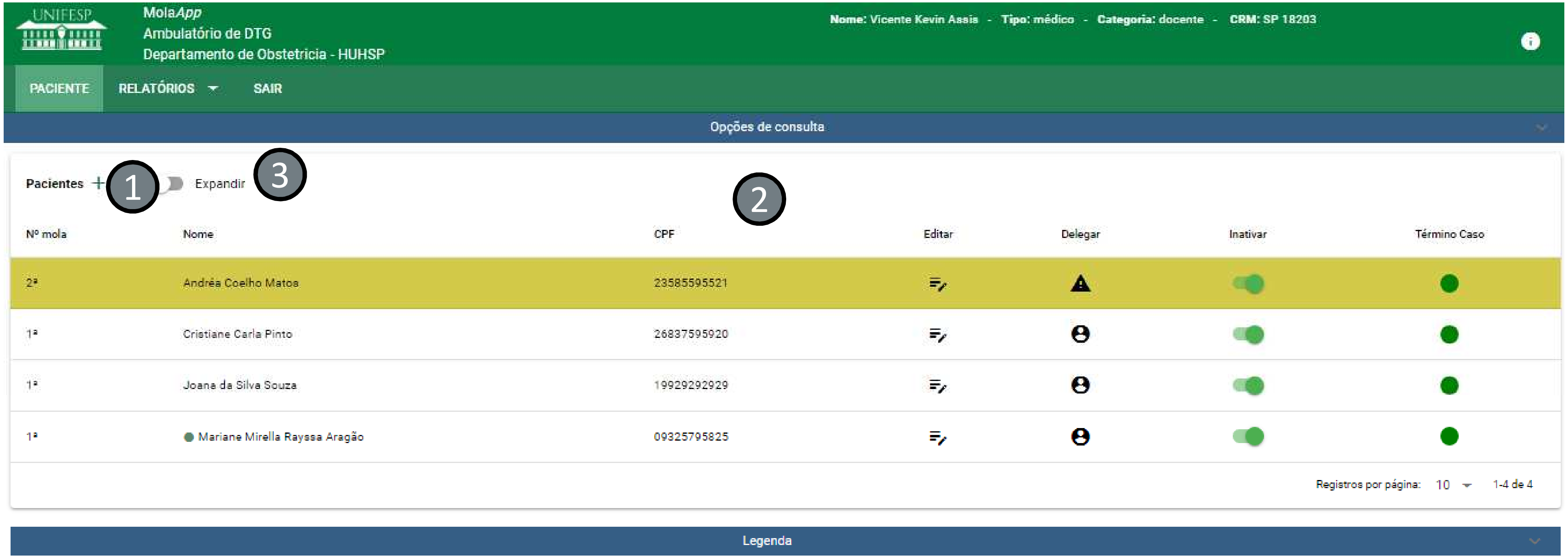

1 – Inclusão de novos pacientes no sistema
2 – Campos de informações gerais sobre os pacientes
3 – Expansão/ocultação de campos de informações gerais sobre os pacientes

MÉDICO

CADASTRO DE PACIENTES NO SISTEMA

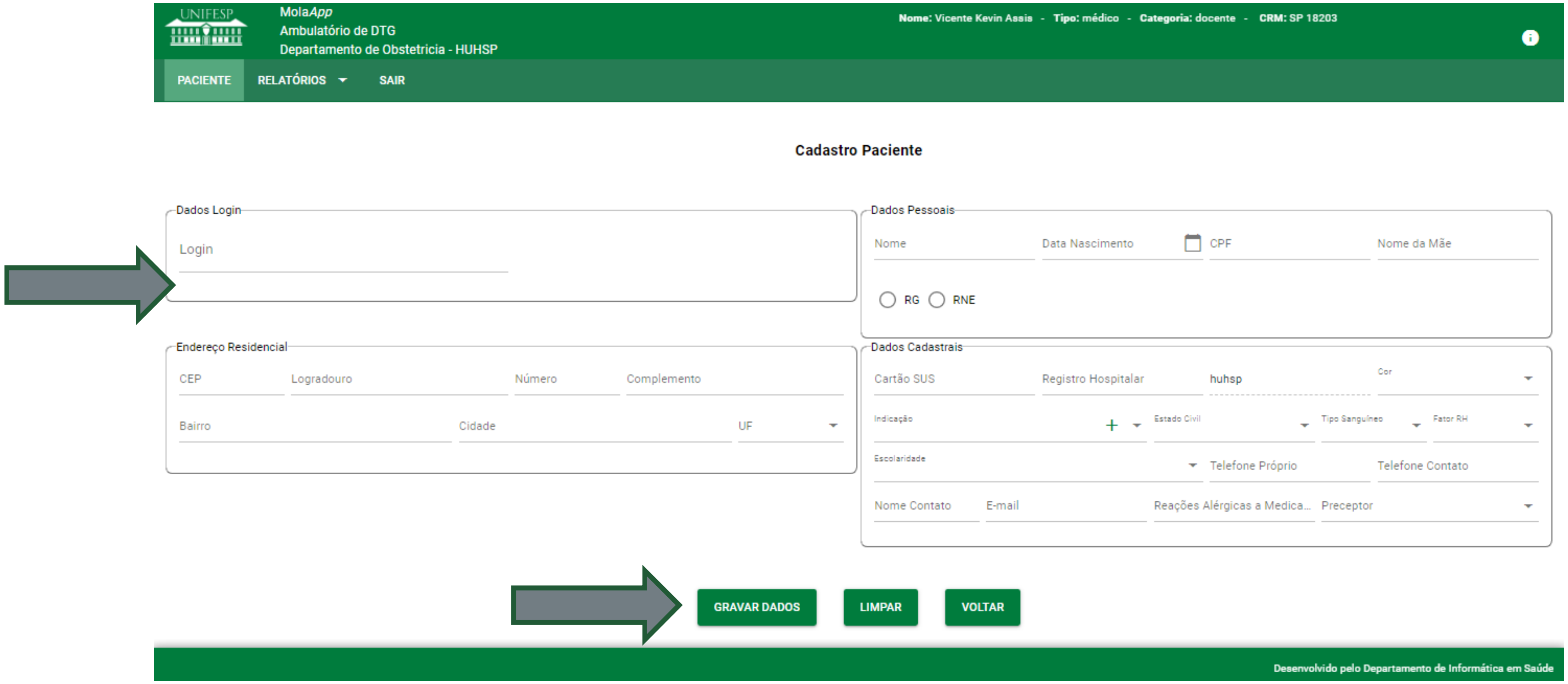

- Ao clicar no sinal "+" na página principal, será aberto o cadastro de pacientes
- Serão inseridos os dados demográficos e o login que o paciente utilizará no aplicativo
- Após preencher o formulário, clicar em "gravar dados"

MÉDICO

INFORMAÇÕES GERAIS SOBRE OS PACIENTES

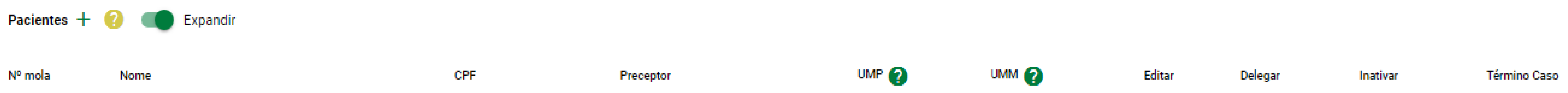

- **N° MOLA:** Identificação se primeira ou segunda ocorrência de Mola na paciente.
- **NOME:** Nome da paciente.
- **CPF:** CPF da paciente.
- **PRECEPTOR*:** Nome do médico responsável pelo acompanhamento longitudinal da paciente.
- **UMP*+:** Data e horário da última mensagem enviada pela paciente.
- **UMM*+:** Data e horário da última mensagem enviada pelo médico.
- **EDITAR:** Inserir/alterar/excluir dados clínicos da paciente.
- **DELEGAR:** Nome do médico responsável pelo atendimento ao paciente.
- **INATIVAR:** Retirar a paciente da lista de casos em acompanhamento.
- **TÉRMINO CASO:** Estado do caso. Pode ser alterado em editar/dados gerais.

*Estes itens aparecem apenas se o “expandir” estiver ativo
+O ícone verde estará na mensagem mais recente: UMP se for o paciente, UMM se o for médico

MÉDICO

FICHA DO PACIENTE

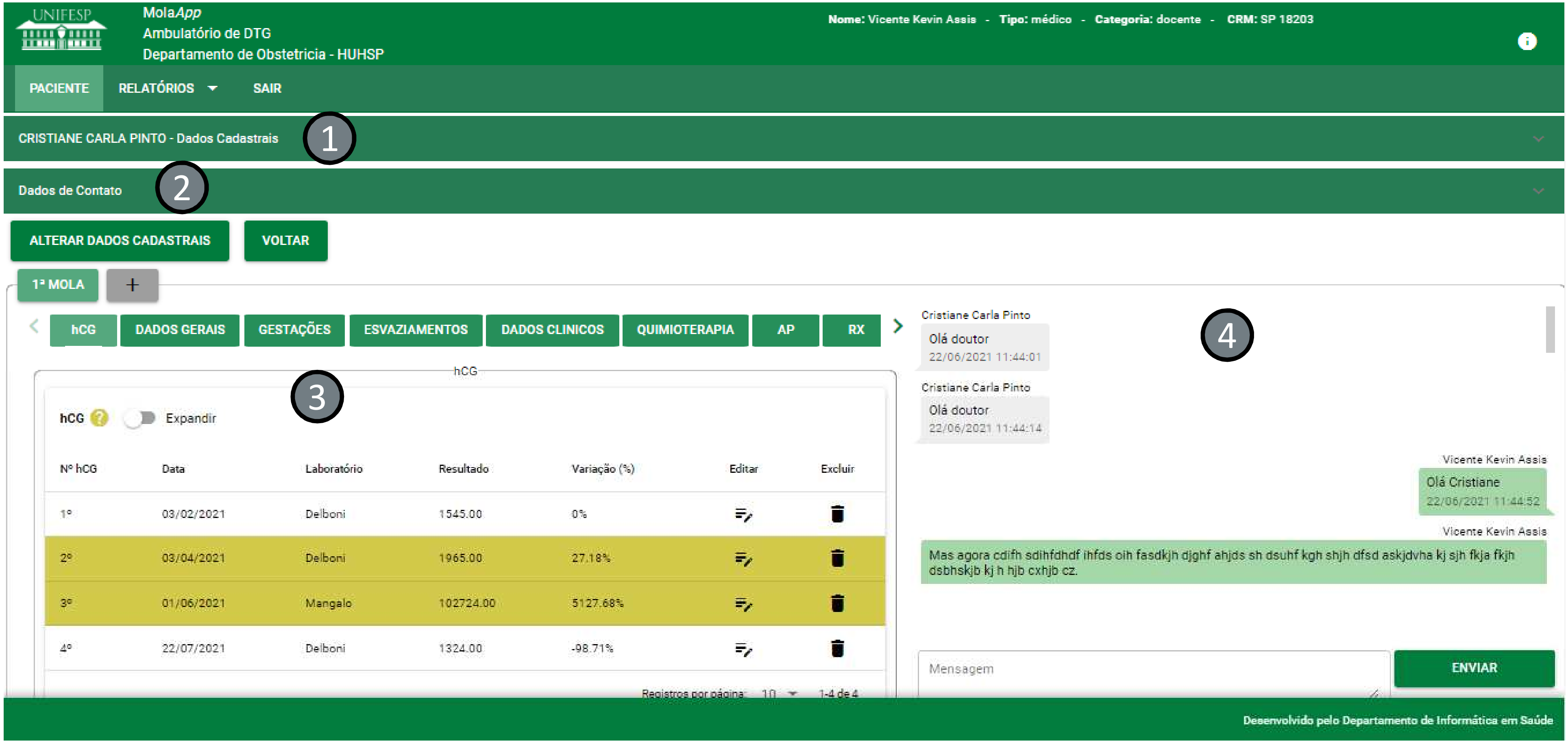

1 – Identificação da paciente

2 – Área de dados cadastrais e clínicos da paciente

3 – Área de visualização/edição das sessões da ficha do paciente

4 – Área de comunicação com a paciente via chat

MÉDICO

ABAS COM DADOS DOS PACIENTES

- **hCG:** Nesta aba será exibido os resultados enviados pelas pacientes via aplicativo ou inserido pelo médico. O profissional poderá editar os resultados. É possível inserir o resultado e enviar uma foto do resultado impresso.
- **DADOS GERAIS:** Status do caso e informações gerais sobre a paciente.
- **GESTAÇÕES:** Dados sobre gestações anteriores.
- **ESVAZIAMENTOS:** Dados sobre o esvaziamento realiado pela paciente
- **DADOS CLÍNICOS:** Resumo de dados clínicos, registrados de forma dicotômica
- **QUIMIOTERAPIA:** Anotações sobre todos os ciclos realizados pela paciente
- **AP:** Resultados de todos os exame anátomo-patológicos realizados pela paciente
- **RX:** Registro dos RX realizados. Pode ser cadastrado pela paciente através do aplicativo ou pelo médico, através do site.
- **ULTRASSOM:** Registro das imagens e /ou laudos dos US realizados. Pode ser cadastrado pela paciente através do aplicativo ou pelo médico, através do site.
- **TOMOGRAFIA:** Registro das imagens e /ou laudos dos TC realizados. Pode ser cadastrado pela paciente através do aplicativo ou pelo médico, através do site.
- **CALENDÁRIO:** Registro da DUM . Pode ser cadastrado pela paciente através do aplicativo ou pelo médico, através do site.

MÉDICO

hCG

hCG | DADOS GERAIS | GESTAÇÕES | ESVAZIAMENTOS | DADOS CLINICOS | QUIMIOTERAPIA | AP | RX | ULTR

hCG

hCG Expandir

Id hCG	Nº hCG	Data	Laboratório	Resultado	Variação (Absoluta)	Variação (%)	Cadastrante	Editar	Excluir
7	1º	03/02/2021	Delboni	1545.00	0	0%	Vicente Kevin Assis		
8	2º	03/04/2021	Delboni	1965.00	420.00	27.18%	Vicente Kevin Assis		
26	3º	01/06/2021	Mangalo	102724.00	100759.00	5127.68%	Vicente Kevin Assis		
21	4º	22/07/2021	Delboni	1324.00	-101400.00	-98.71%	Vicente Kevin Assis		

Registros por página: 10 1-4 de 4

NOVO EXAME

MÉDICO

hCG

- **ID HCG:** Identificação do sistema para o exame.
- **Nº HCG:** Ordem de registro do exame hCG.
- **RESULTADO:** Valor obtido no exame de hCG
- **VARIAÇÃO (ABSOLUTA)*:** A diferença entre o último e o penúltimo resultado de hCG.
- **VARIAÇÃO (%):** Variação relativa entre o último e o penúltimo resultado de hCG..
- **CADASTRANTE*:** Nome do responsável pelas informações do exame.
- **EXCLUIR:** Deletar exame registrado.
- **NOVO EXAME:** Cadastrar no registro de exame pelo sistema.

*Estes itens aparecem apenas se o “expandir” estiver ativo

MÉDICO

hCG – CADASTRO DE NOVO EXAME PELO SISTEMA

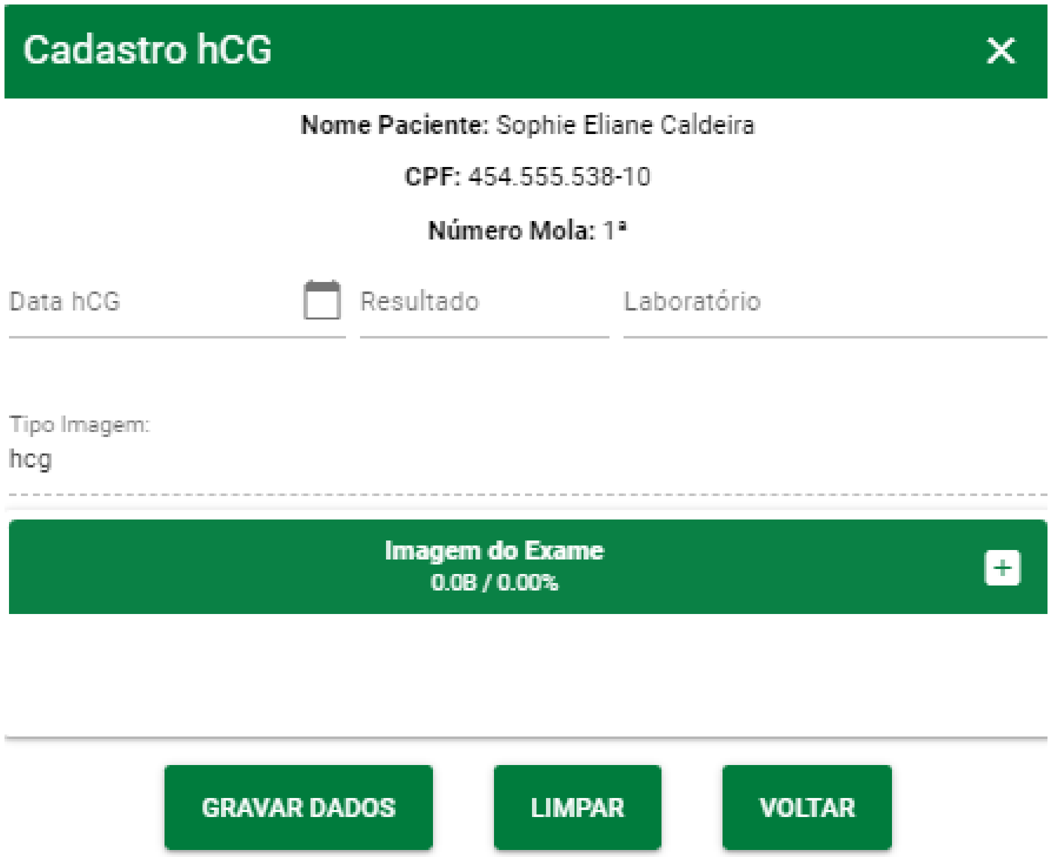

MÉDICO

DADOS GERAIS

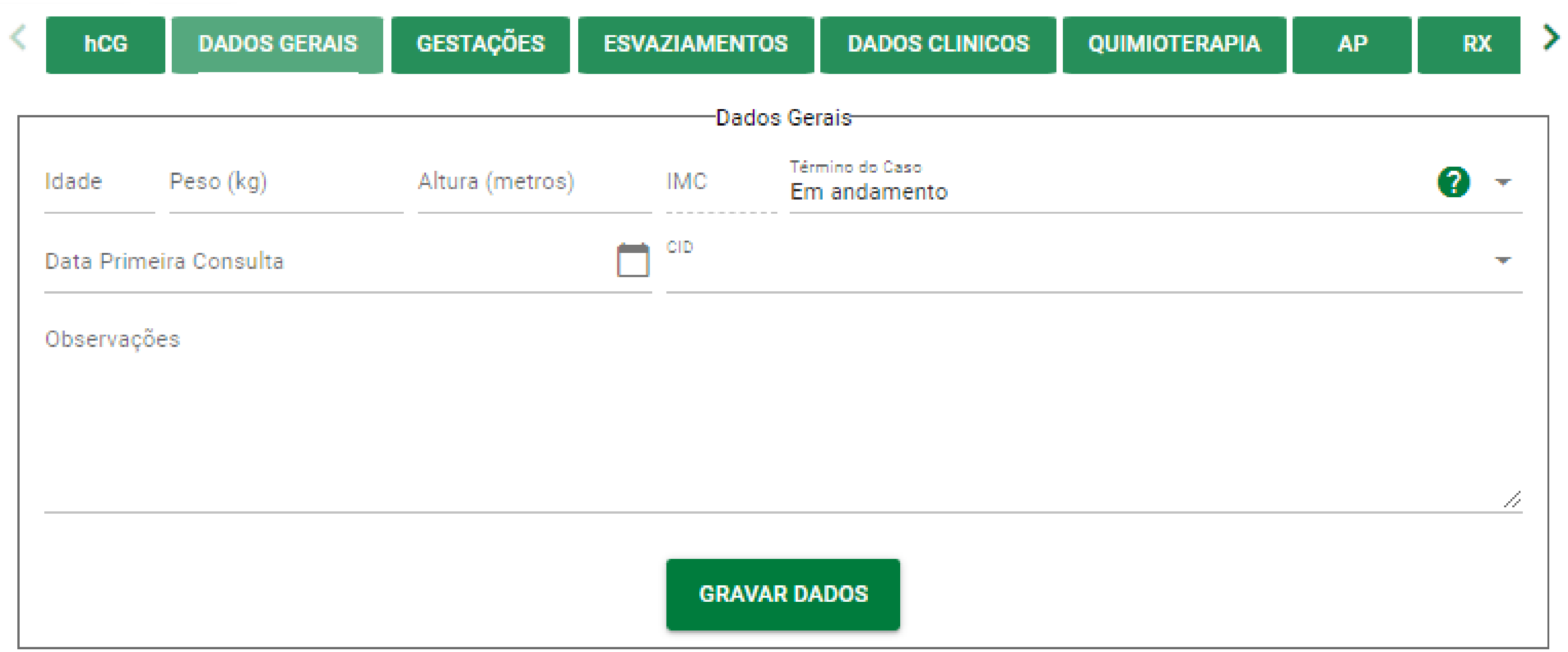

MÉDICO

GESTAÇÕES

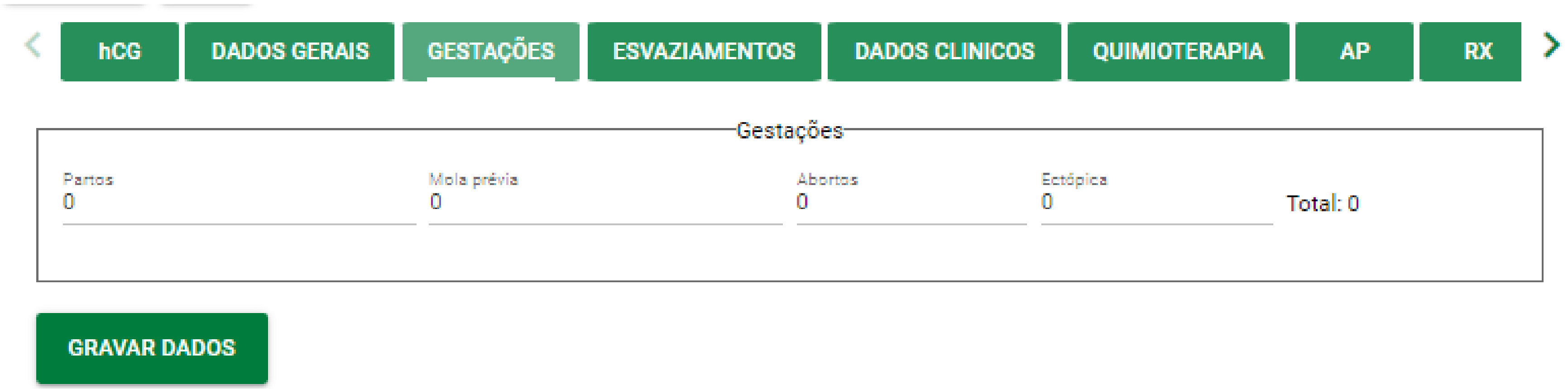

MÉDICO

ESVAZIAMENTOS

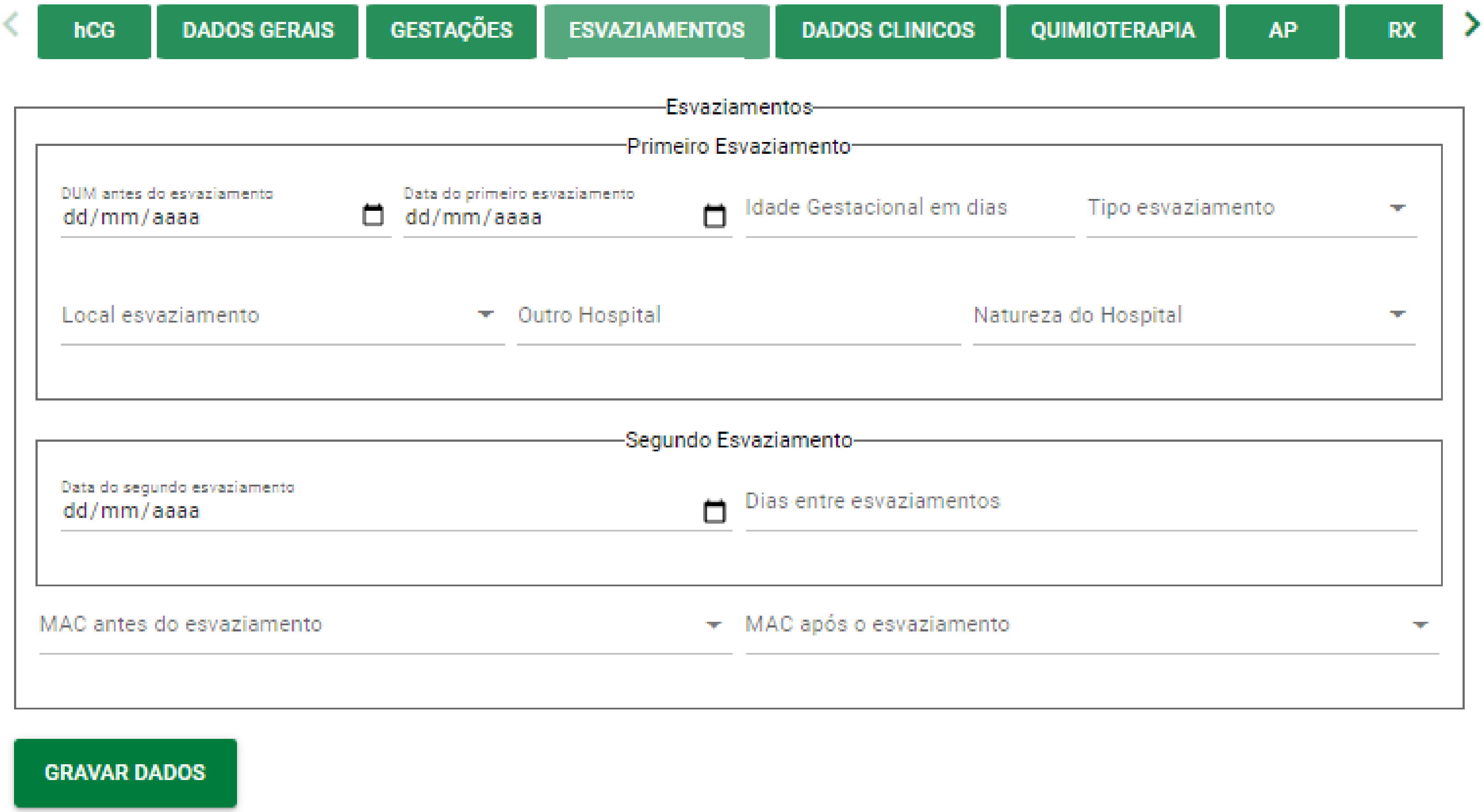

MÉDICO

DADOS CLÍNICOS

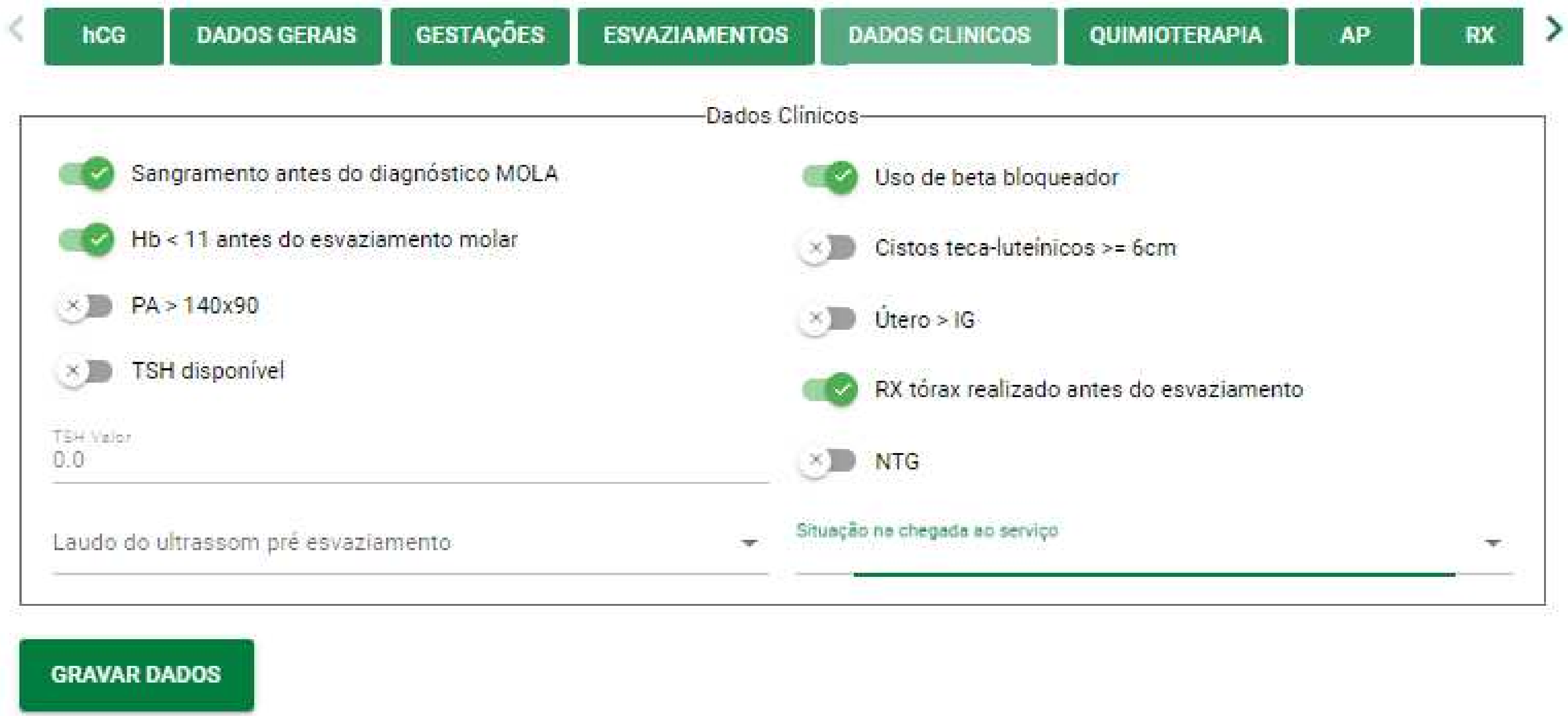

Se o item estiver verde, ele está selecionado e marcado como SIM

MÉDICO

QUIMIOTERAPIA

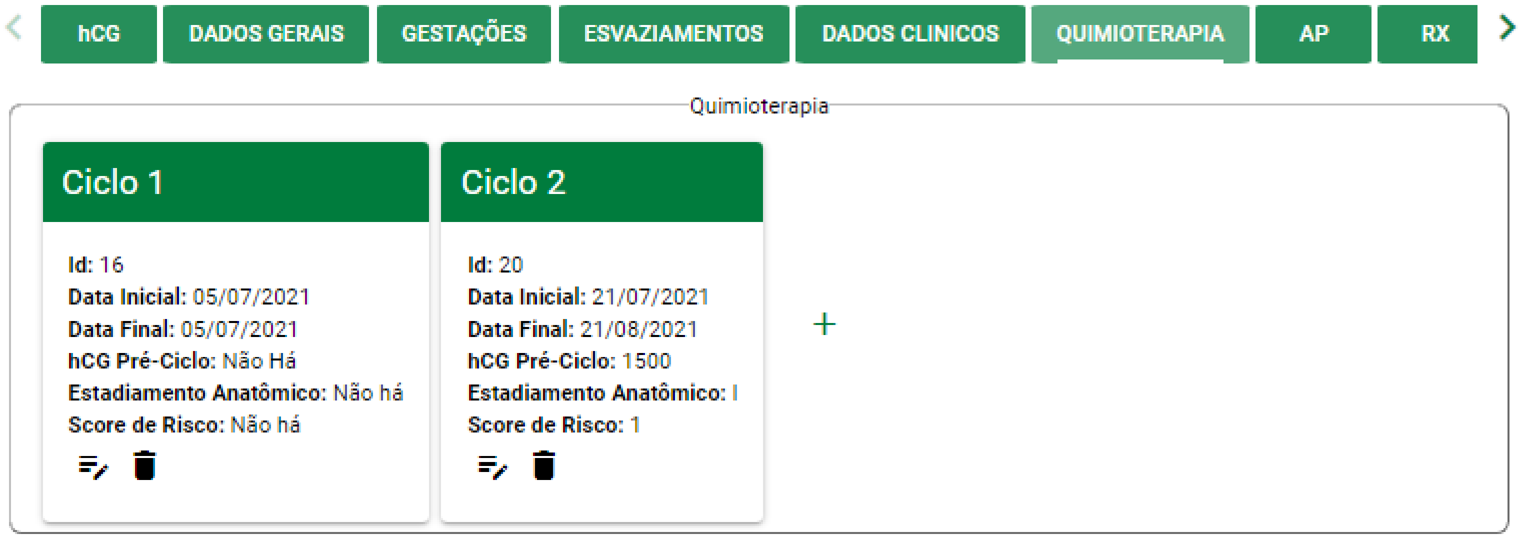

O ciclo de quimioterapia é visualizado em forma de cartão

Para incluir novo ciclo, basta clicar no sinal “+”

MÉDICO

QUIMIOTERAPIA – CADASTRO DE CICLOS

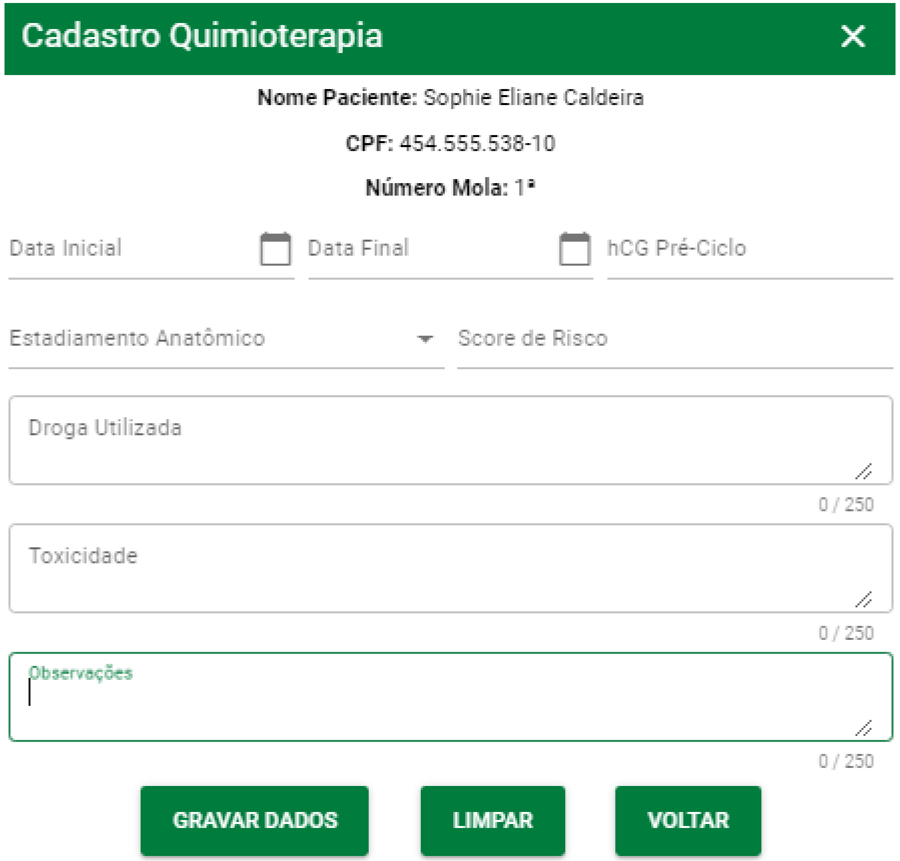

MÉDICO

ANATOMIA PATOLÓGICA

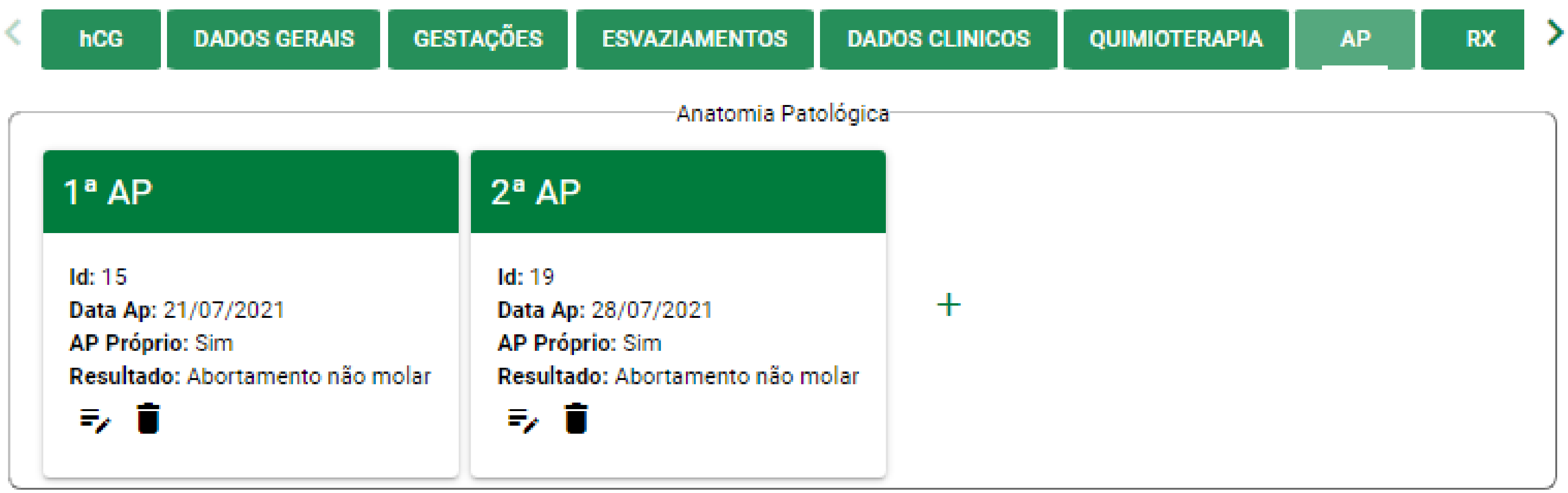

O exame de anatomia patológica é visualizado em forma de cartão

Para editá-lo, no ícone inferior à esquerda. A lixeira exclui o registro

Para incluir novo ciclo, basta clicar no sinal “+”

MÉDICO

ANATOMIA PATOLÓGICA – CADASTRO DE EXAMES

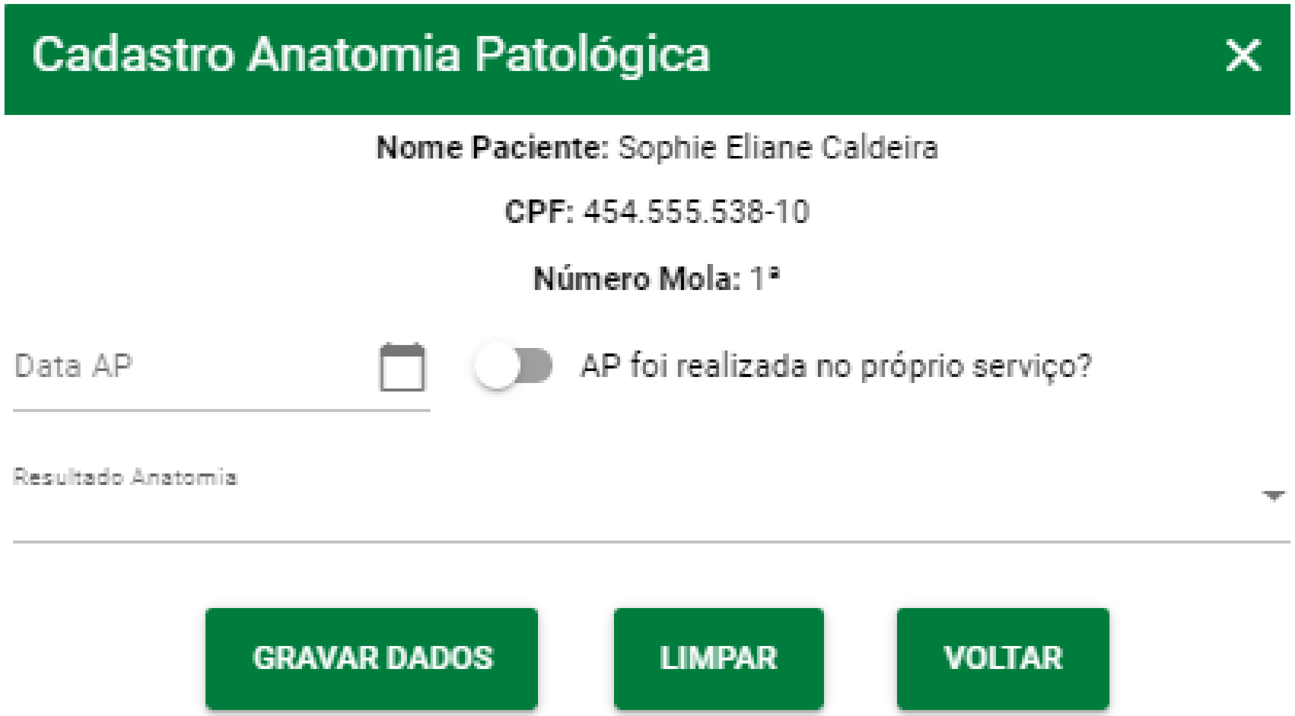

MÉDICO

RAIO-X

hCG | DADOS GERAIS | GESTAÇÕES | ESVAZIAMENTOS | DADOS CLINICOS | QUIMIOTERAPIA | AP | RX

Raio-X

1º Raio-X

Id: 14
Tipo: RX
Data do Exame: 13/07/2021

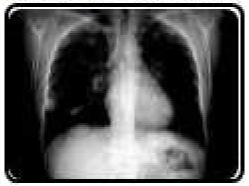

Cadastrante: Sophie Eliane Caldeira

2º Raio-X

Id: 16
Tipo: RX
Data do Exame: 17/07/2021

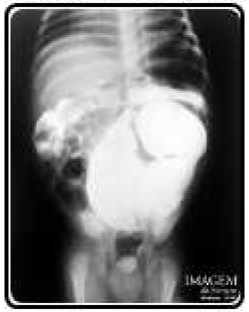

Cadastrante: Isaac Henry Moreira

+

A imagem de raio-x é separada em forma de cartão.
Para visualizá-lo/editá-lo, no ícone inferior à esquerda. A lixeira exclui o registro
Para incluir nova imagem, basta clicar no sinal “+”

MÉDICO

RAIO-X – CADASTRO DE EXAME

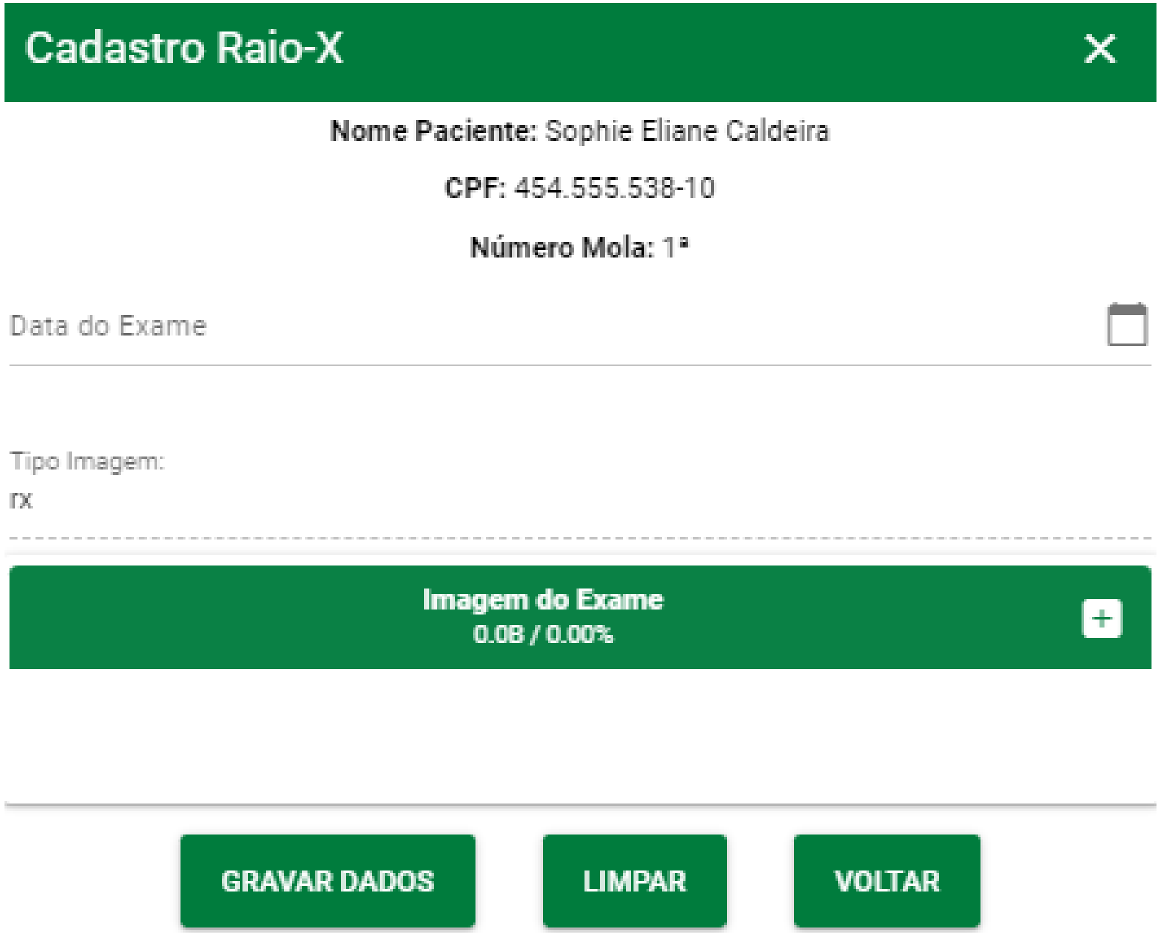

MÉDICO

ULTRASSOM

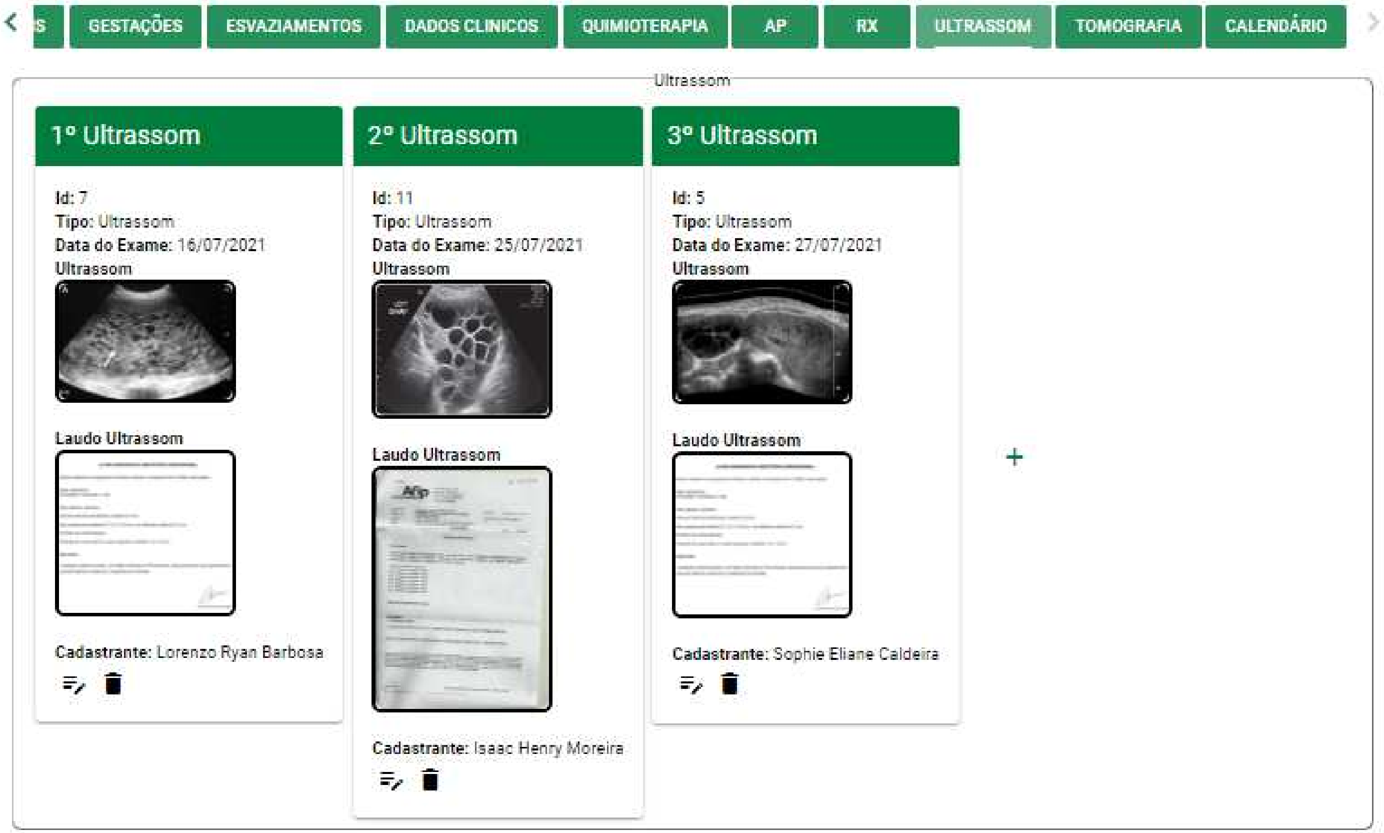

O laudo/imagem de ultrassom são separados em forma de cartão.
Para visualizá-lo/editá-lo, no ícone inferior à esquerda. A lixeira exclui o registro
Para incluir novo laudo/imagem, basta clicar no sinal "+"

MÉDICO

ULTRASSOM – CADASTRO DE LAUDO/EXAME

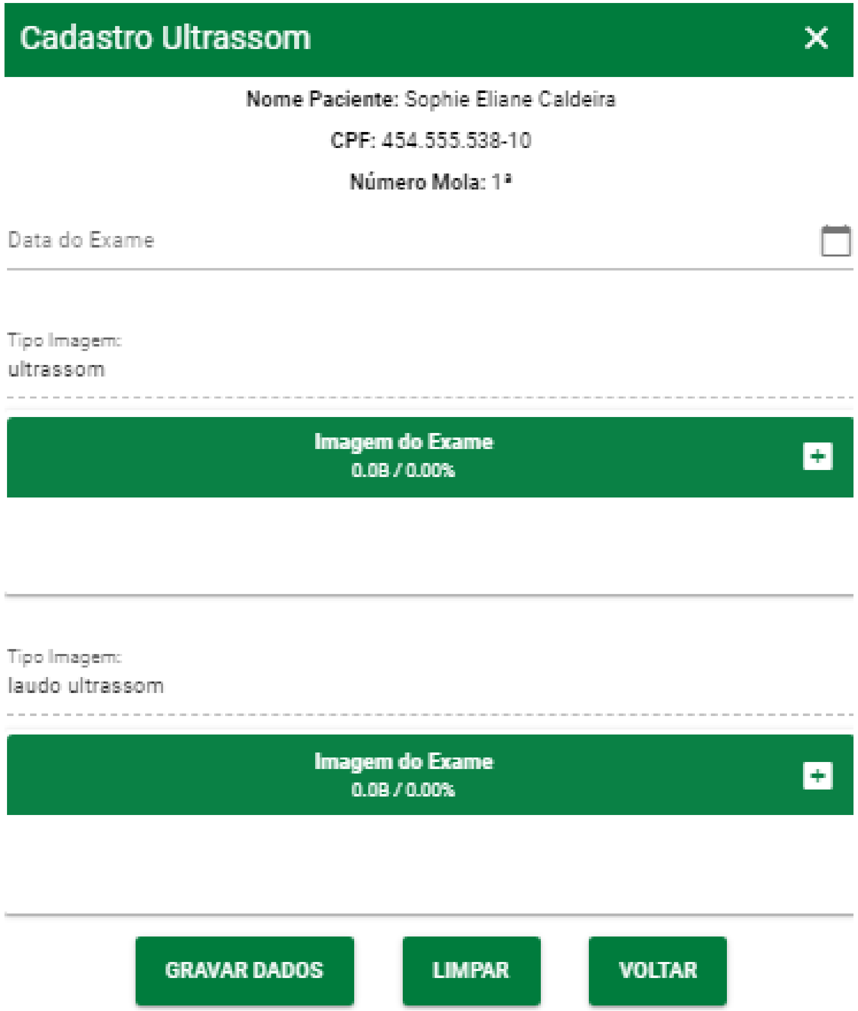
Cadastro Ultrassom

Nome Paciente: Sophie Eliane Caldeira

CPF: 454.555.538-10

Número Mola: 1ª

Data do Exame

Tipo Imagem:
ultrassom

Imagem do Exame
0.0B / 0.00%

Tipo Imagem:
laudo ultrassom

Imagem do Exame
0.0B / 0.00%

GRAVAR DADOS | LIMPAR | VOLTAR

MÉDICO

TOMOGRAFIA

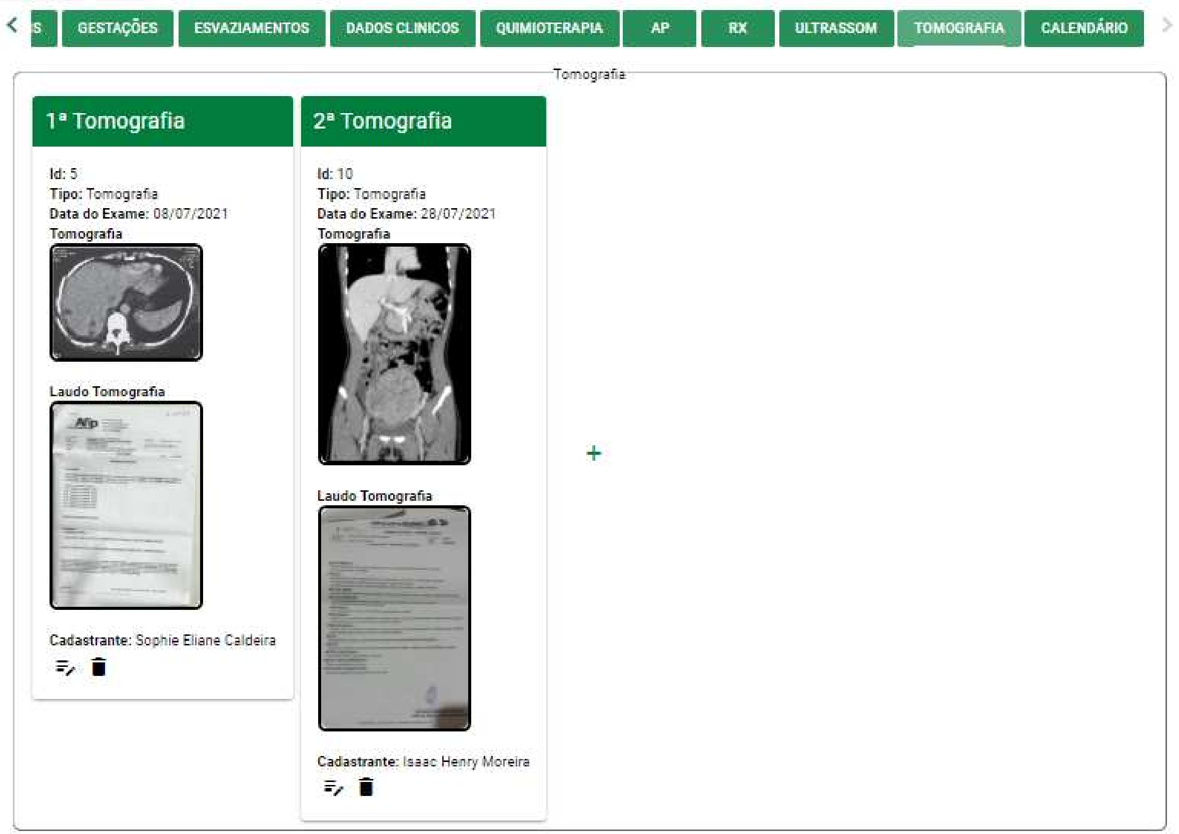

O laudo/imagem de tomografia são separados em forma de cartão.
Para visualizá-lo/editá-lo, no ícone inferior à esquerda. A lixeira exclui o registro
Para incluir novo laudo/imagem, basta clicar no sinal “+”

MÉDICO

TOMOGRAFIA – CADASTRO DE LAUDO/IMAGEM

Cadastro Tomografia ✕

Nome Paciente: Sophie Eliane Caldeira

CPF: 454.555.538-10

Número Mola: 1ª

Data do Exame

Tipo Imagem:
tomografia

Imagem do Exame
0.0B / 0.00%

Tipo Imagem:
laudo tomografia

Imagem do Exame
0.0B / 0.00%

GRAVAR DADOS | LIMPAR | VOLTAR

MÉDICO

CALENDÁRIO

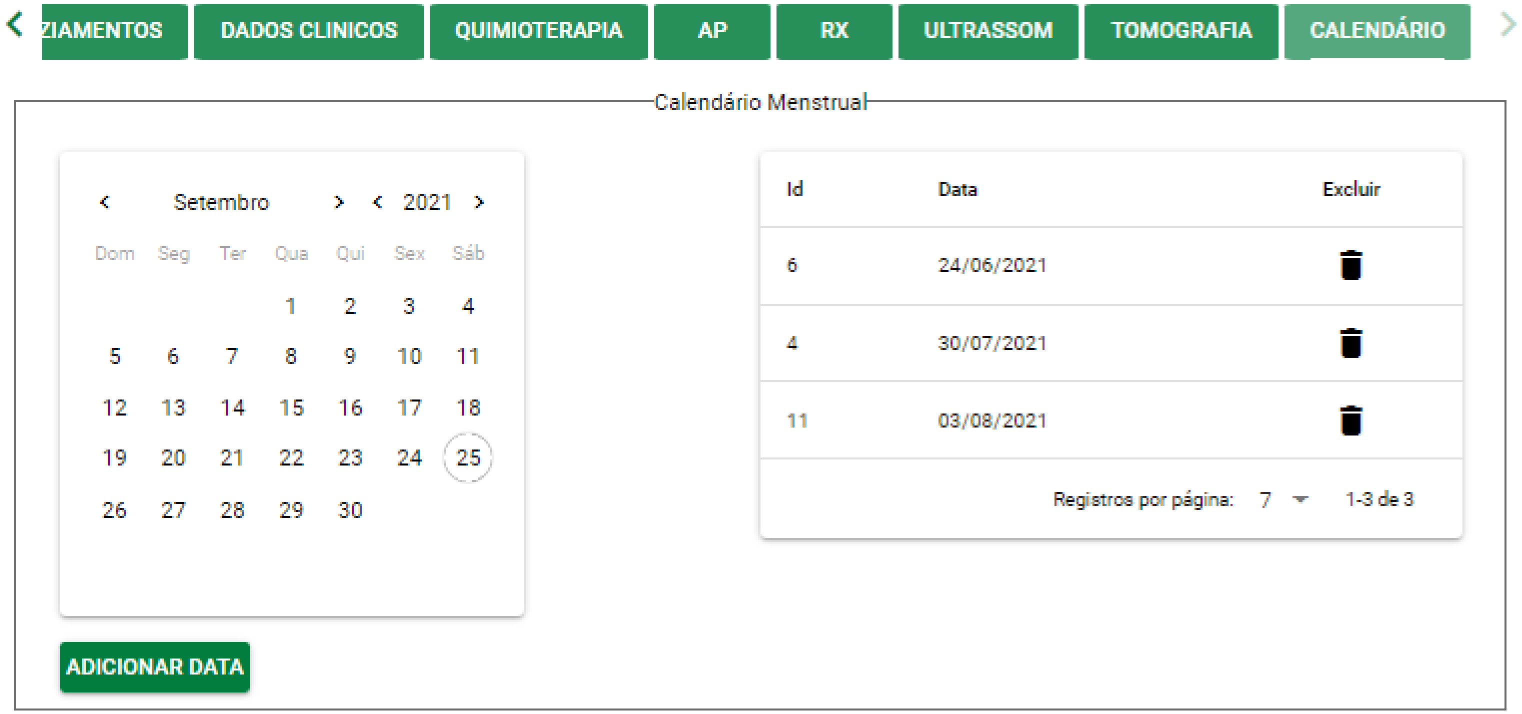

Calendário para visualização da DUM.
O cadastro é realizado pela paciente ou pelo médico responsável

MÉDICO

CHAT

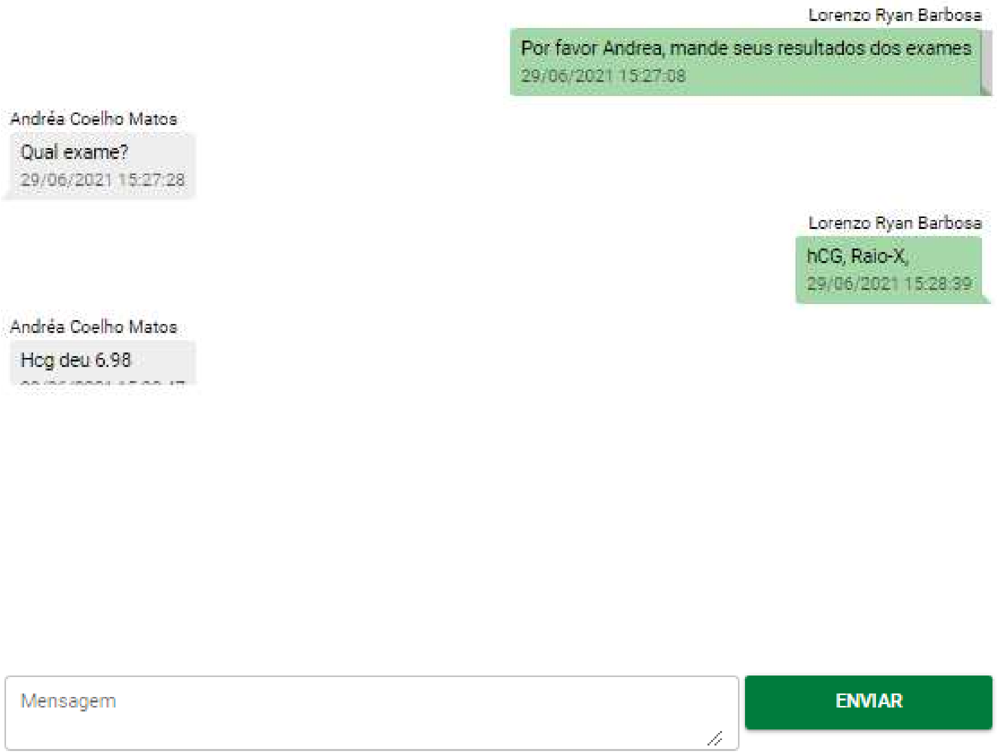

Este espaço é reservado para a conversa entre o médico e o paciente.
O paciente só receberá mensagem se estiver cadastrado no sistema.
Não é possível enviar mídia, apenas texto.

Logotipo utilizado no aplicativo

Universidade Federal de São Paulo

Hospital Universitário / Hospital São Paulo

Ambulatório de Doença Trofoblástica

Gestacional

PACIENTE

Universidade Federal de São Paulo
Hospital Universitário / Hospital São Paulo
Ambulatório de Doença Trofoblástica Gestacional

2021

PACIENTE

- **O acesso será exclusivamente por aplicativo.**
- **Esta versão está disponível apenas para Android.**
- **Você receberá o aplicativo via whatsapp enviado pelo médico que a atende.**
- **Ele a auxiliará na instalação do aplicativo em seu celular.**
- **O aplicativo dispõe de duas áreas:**
  - ✓ **Informações gerais sobre a DTG**
  - ✓ **Área reservada para o seu tratamento, liberada após cadastro que será realizado por seu médico**

PACIENTE

TELA INICIAL - INFORMAÇÕES

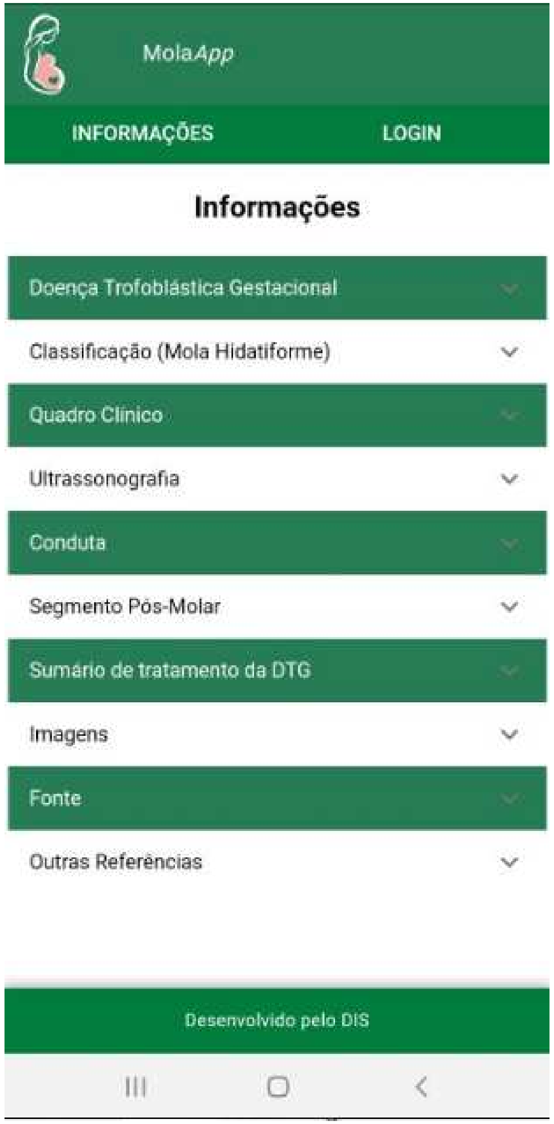

- ✓ **Você encontrará neste espaço informações resumidas sobre as principais dúvidas referentes à Doença Trofoblástica Gestacional.**
- ✓ **Basta você ter o app instalado em seu celular ou tablet**
- ✓ **Clique na seta para baixo, ao lado do título, para que apareça um texto explicativo**

PACIENTE

TELA INICIAL - LOGIN

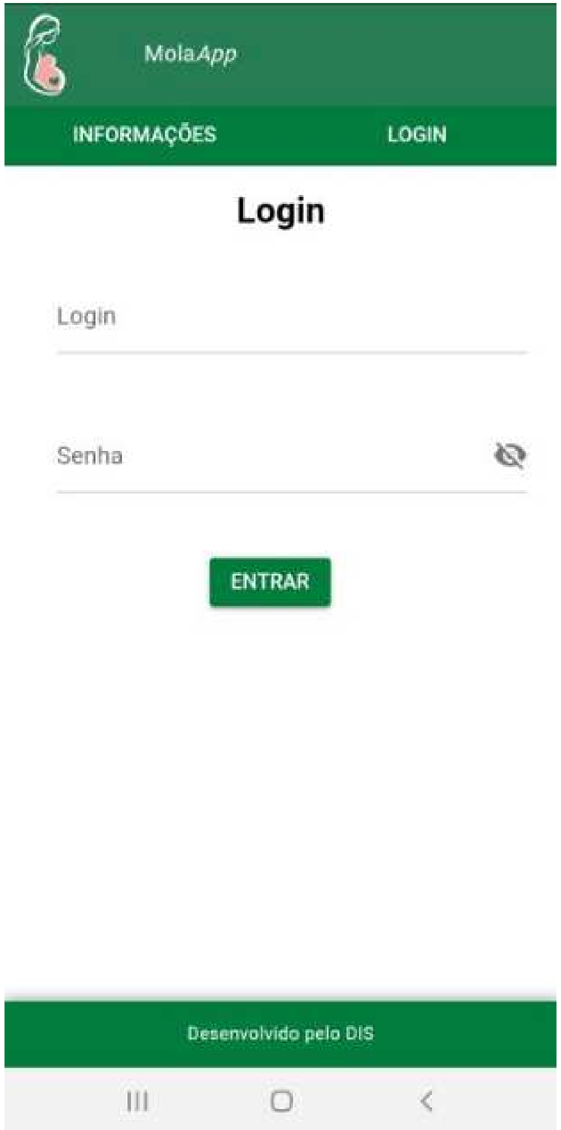

- ✓ O seu login e senha serão fornecidos pelo seu médico pois ele fará o cadastro no sistema.
- ✓ Somente pacientes acompanhadas no ambulatório poderão utilizar este serviço exclusivo.
- ✓ No primeiro acesso, o login e a senha serão iguais

PACIENTE

MENU PRINCIPAL

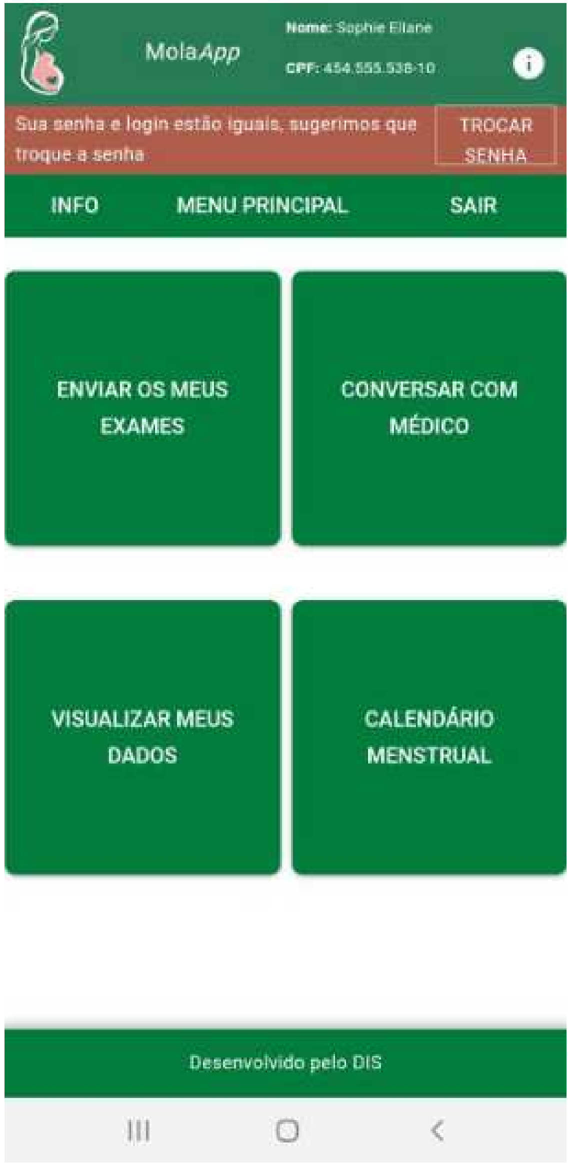

- ✓ **Nesta área está todas funções que você poderá acessar durante o seu tratamento.**
- ✓ **É possível enviar os resultados dos exames de hCG, raio-X, ultrassonografia e tomografia**
- ✓ **Enviar mensagem para o seu médico**
- ✓ **Visualizar os exames enviados**
- ✓ **Anotar a data da última menstruação**

PACIENTE

ALTERAR SENHA

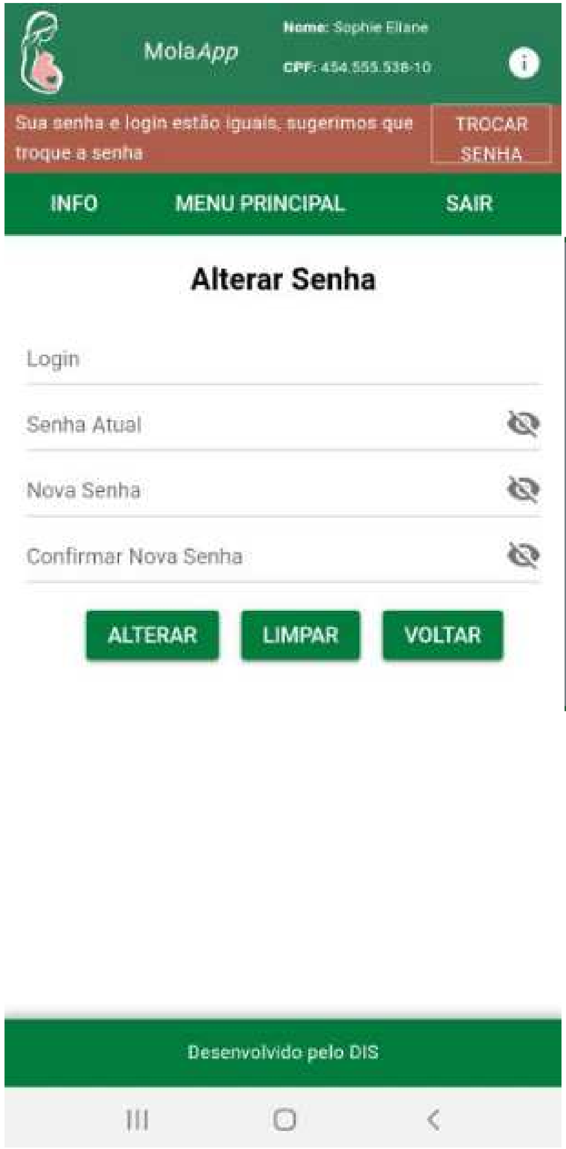

- ✓ No primeiro acesso ao aplicativo, aparecerá esta faixa para você alterar sua senha

- ✓ A qualquer momento, quando você clicar em TROCAR SENHA, aparecerá uma tela como essa para você.
- ✓ Preencha os dados e depois clique em ALTERAR

PACIENTE

MENU PRINCIPAL

- ✓ Quando precisar enviar quaisquer exames para o seu médico, clique em **ENVIAR OS MEUS EXAMES**

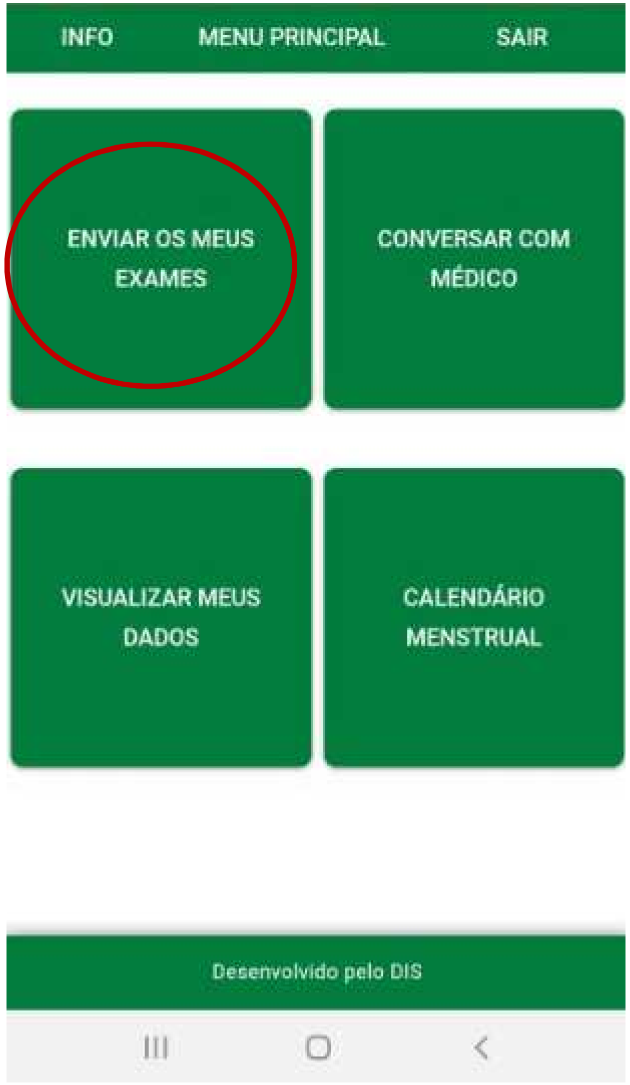

PACIENTE

ENVIAR OS MEUS EXAMES

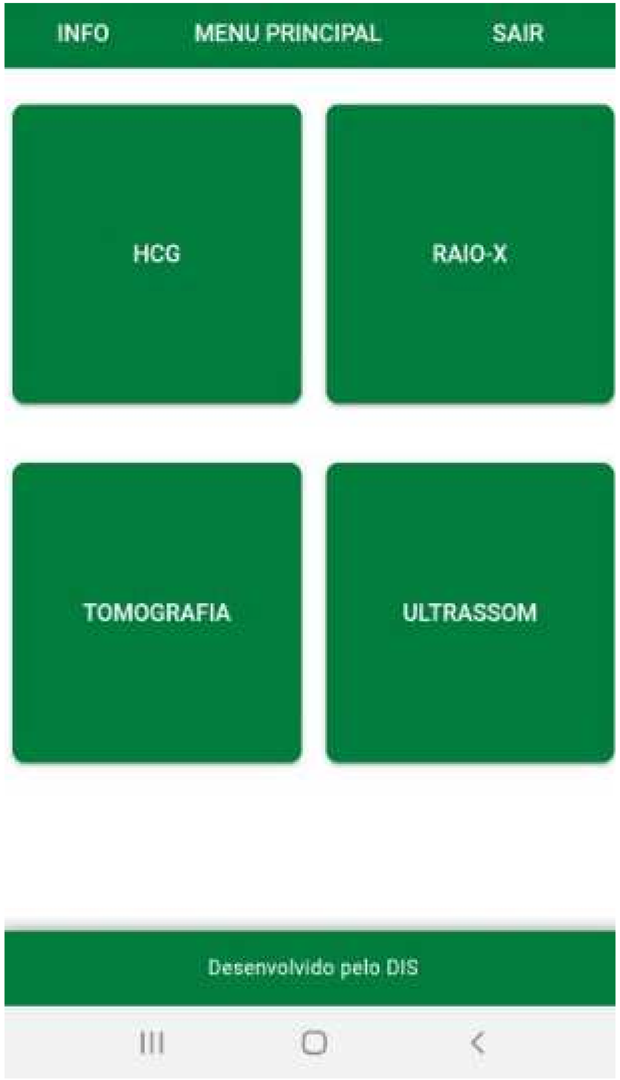

- ✓ **Abrirá uma tela com as opções de exame que você pode enviar.**
- ✓ **Aperte em qual deseja enviar**

PACIENTE

ENVIAR OS MEUS EXAMES - HCG

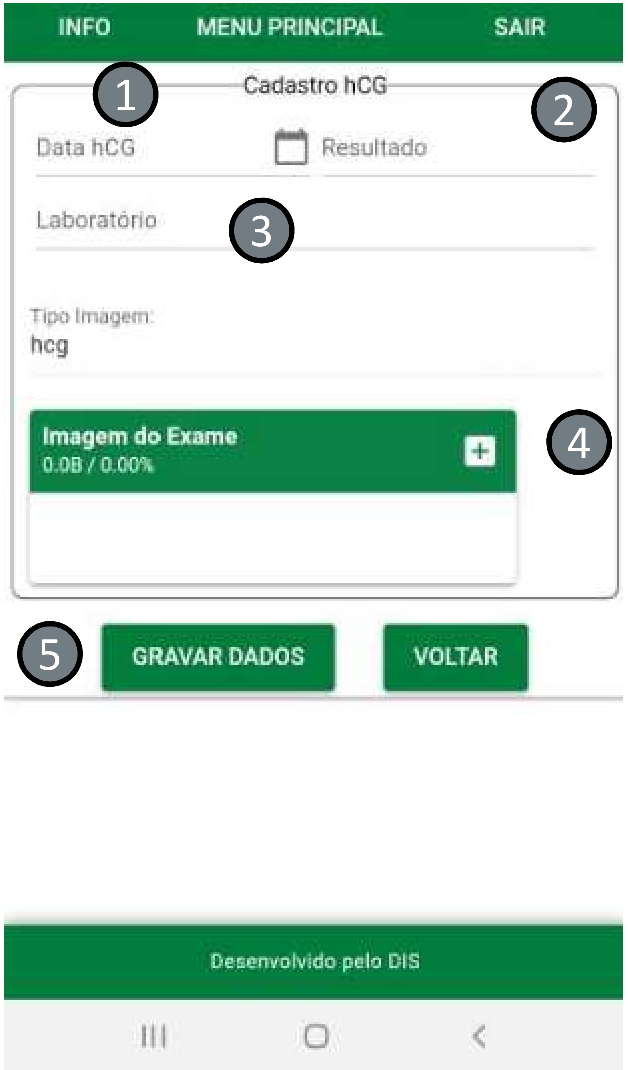

1) **Digitar a data que realizou o exame**

2) **Digitar o resultado do exame**

3) **Digitar em qual laboratório colheu o exame**

4) **Enviar para o seu médico uma foto do resultado do exame. Primeiro tire uma foto e depois localize-a em seu celular**

5) **Apertar em GRAVAR DADOS**

PACIENTE

ENVIAR OS MEUS EXAMES – RAIO-X

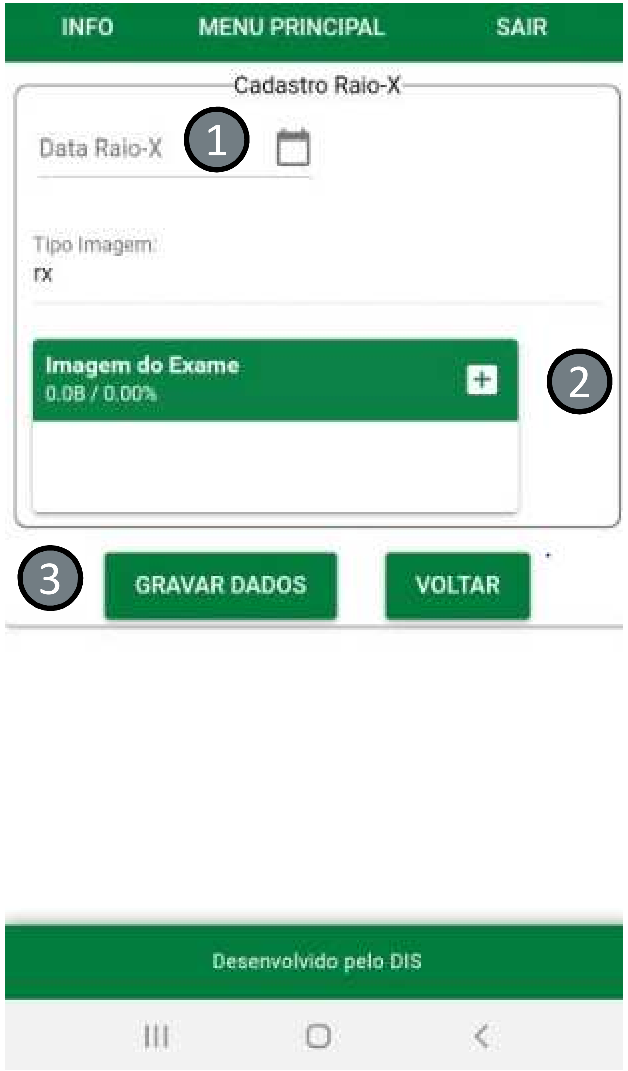

1) Digitar a data que realizou o exame

2) Enviar para o seu médico uma foto do raixo-X.

   Primeiro tire uma foto e depois localize-a em seu celular

3) Apertar em GRAVAR DADOS

PACIENTE

ENVIAR OS MEUS EXAMES - TOMOGRAFIA

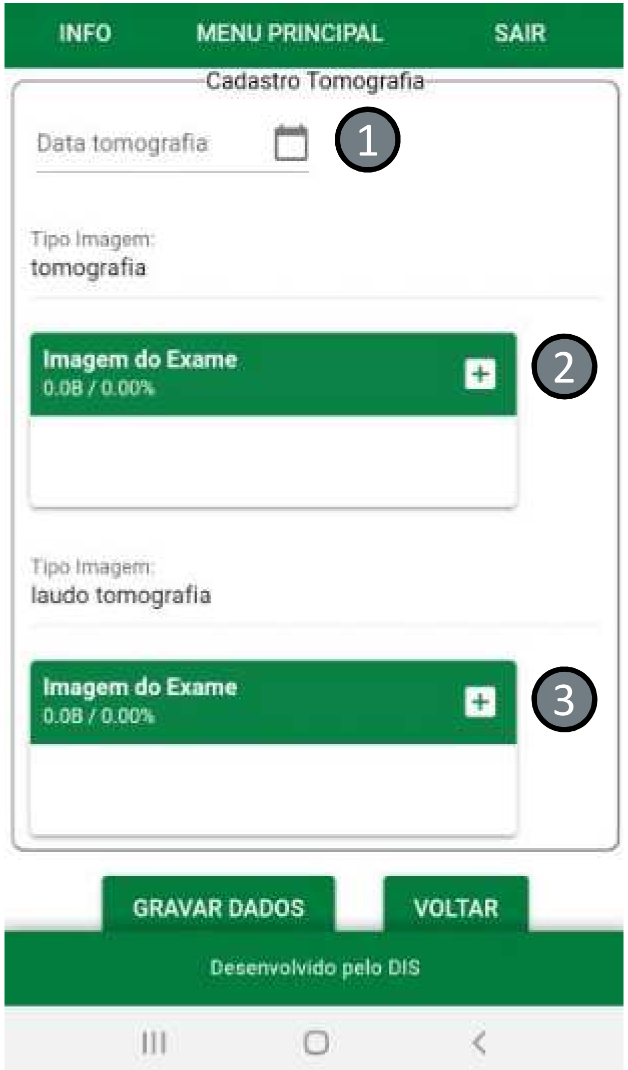

1) Digitar a data que realizou o exame
2) Enviar para o seu médico uma foto da tomografia. Primeiro tire uma foto e depois localize-a em seu celular
3) Enviar para o seu médico uma foto do laudo da tomografia. Primeiro tire uma foto e depois localize-a em seu celular
4) Apertar em GRAVAR DADOS

PACIENTE

ENVIAR OS MEUS EXAMES - ULTRASSOM

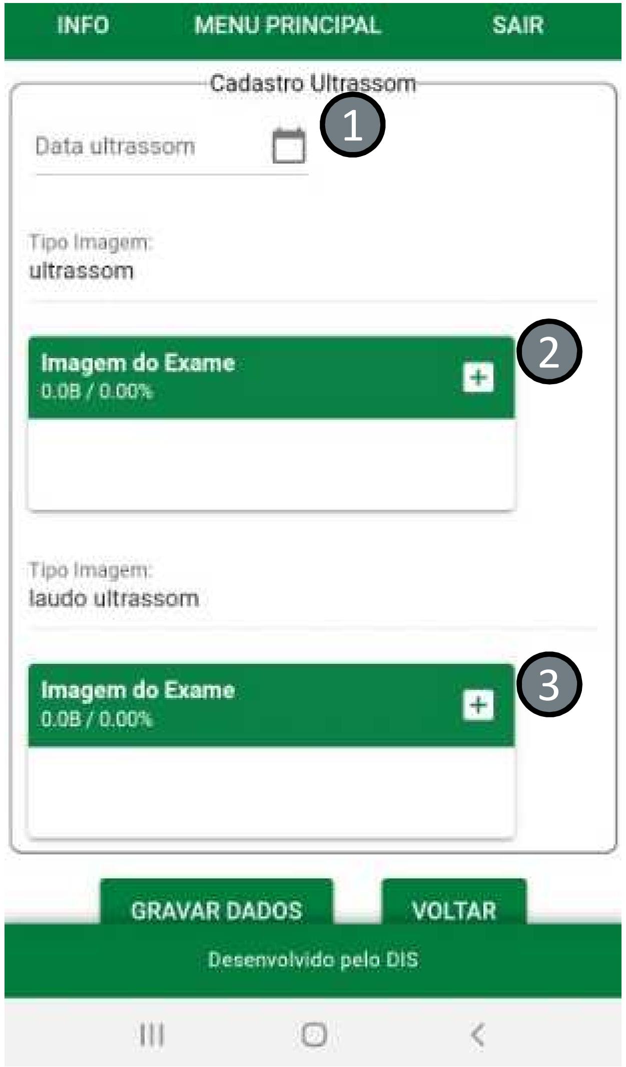

1) **Digitar a data que realizou o exame**

2) **Enviar para o seu médico uma foto do ultrassom. Primeiro tire uma foto e depois localize-a em seu celular**

3) **Enviar para o seu médico uma foto do laudo do ultrassom. Primeiro tire uma foto e depois localize-a em seu celular**

4) **Apertar em GRAVAR DADOS**

PACIENTE

CONVERSAR COM O MÉDICO

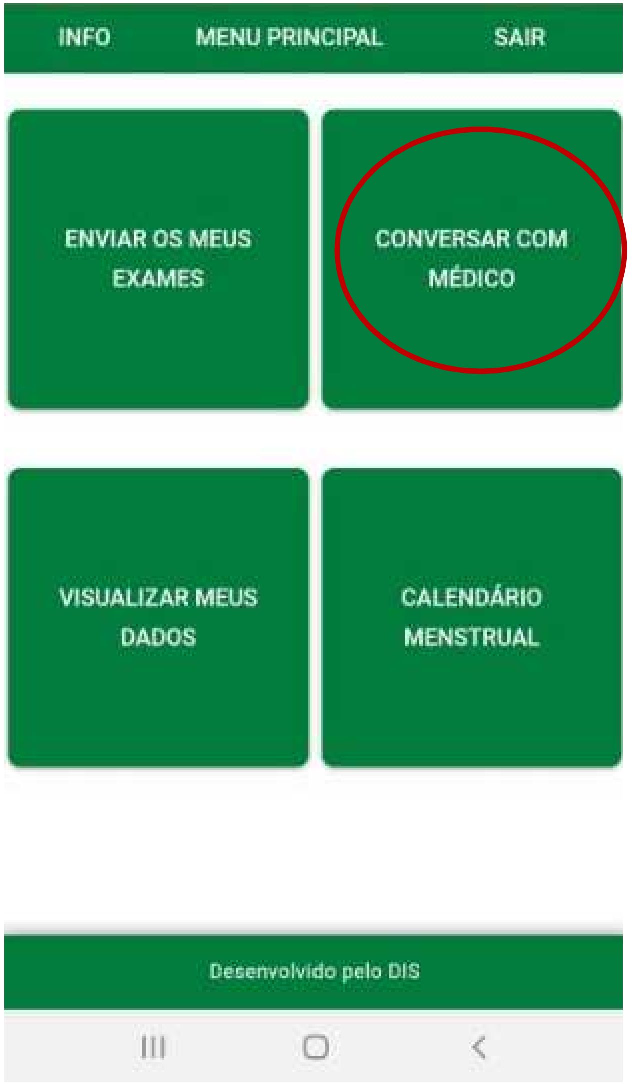

- ✓ **Quando precisar fazer alguma pergunta ou informar algo importante ao seu médico, clique em CONVERSAR COM MÉDICO**

PACIENTE

CONVERSAR COM O MÉDICO

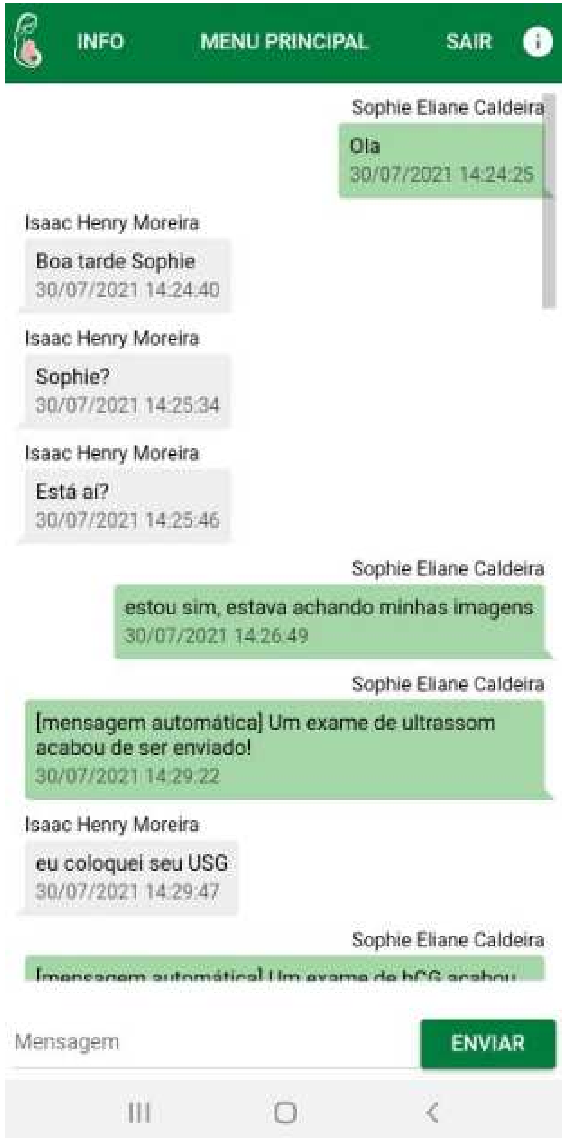

- ✓ Nesta área ficará registrada toda conversa entre você e seu médico.
- ✓ Aparecerá o nome, a mensagem e o horário que foi enviado
- ✓ O seu médico também poderá enviar mensagem para você quando for necessário.

- ✓ Digite sua mensagem e aperte em ENVIAR

PACIENTE

VISUALIZAR MEUS DADOS

✓ Quando quiser ver todos os exames que enviou para o seu médico através do aplicativo, aperte em **VISUALIZAR MEUS DADOS**

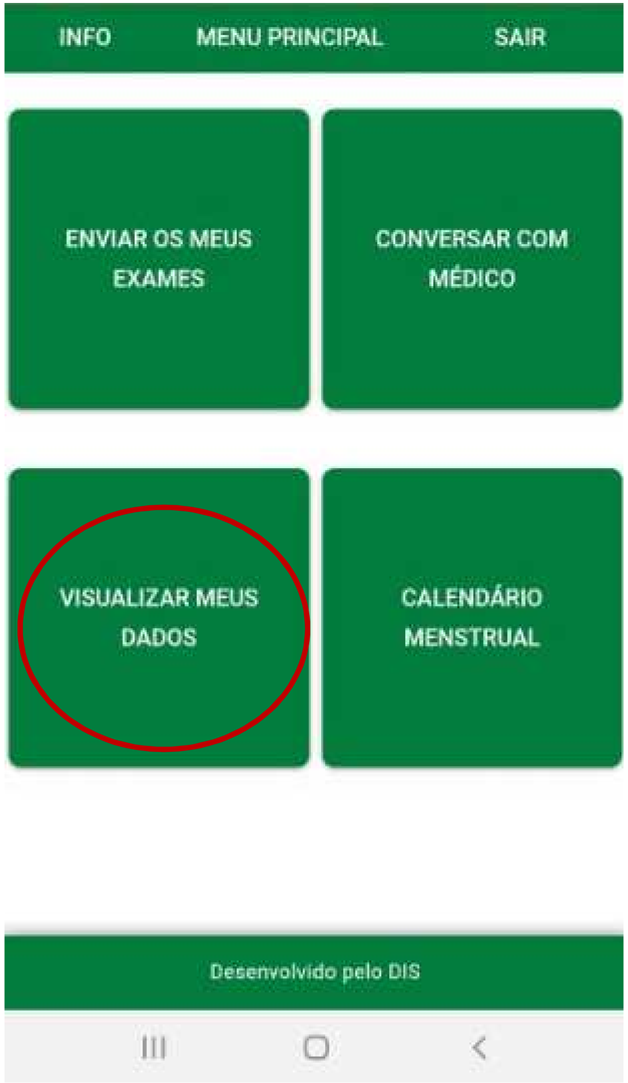

PACIENTE

VISUALIZAR MEUS DADOS

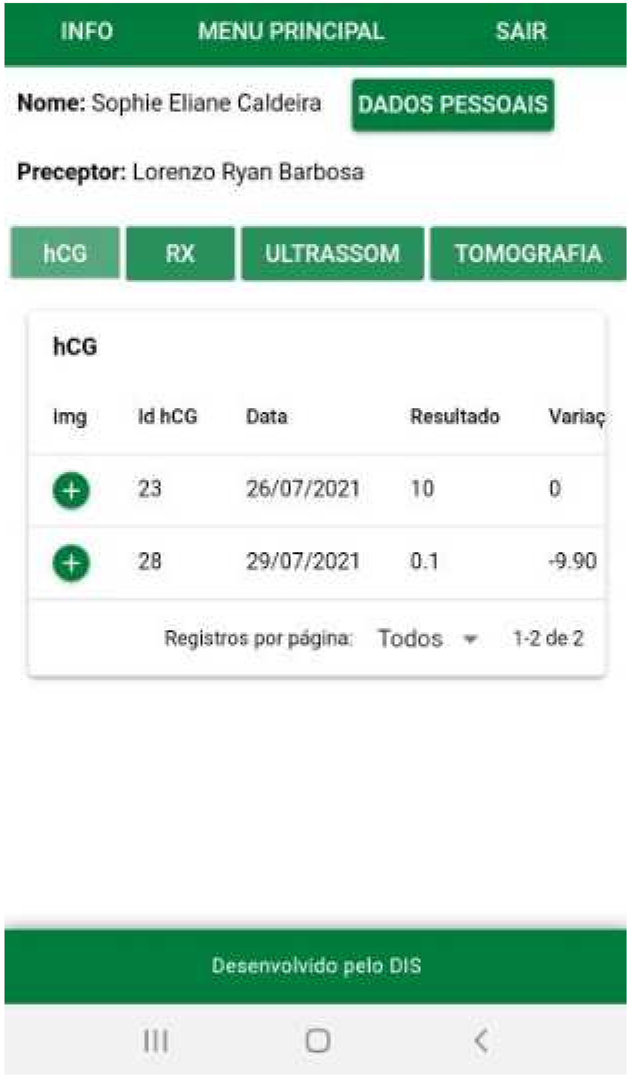

✓ Ao clicar nas abas hCG, RX, ULTRASSOM ou TOMOGRAFIA, aparecerá na parte debaixo da tela as informações sobre os exames enviados.

PACIENTE

CALENDÁRIO MENSTRUAL

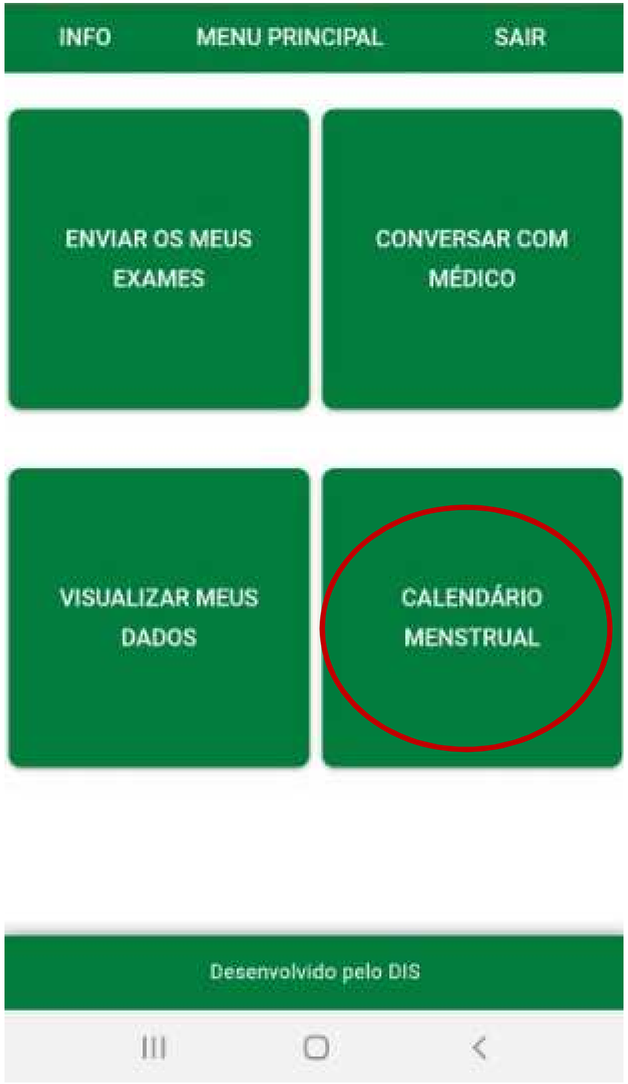

✓ **Aperte aqui para anotar a data de sua última menstruação: CALENDÁRIO MENSTRUAL**

PACIENTE

CALENDÁRIO MENSTRUAL

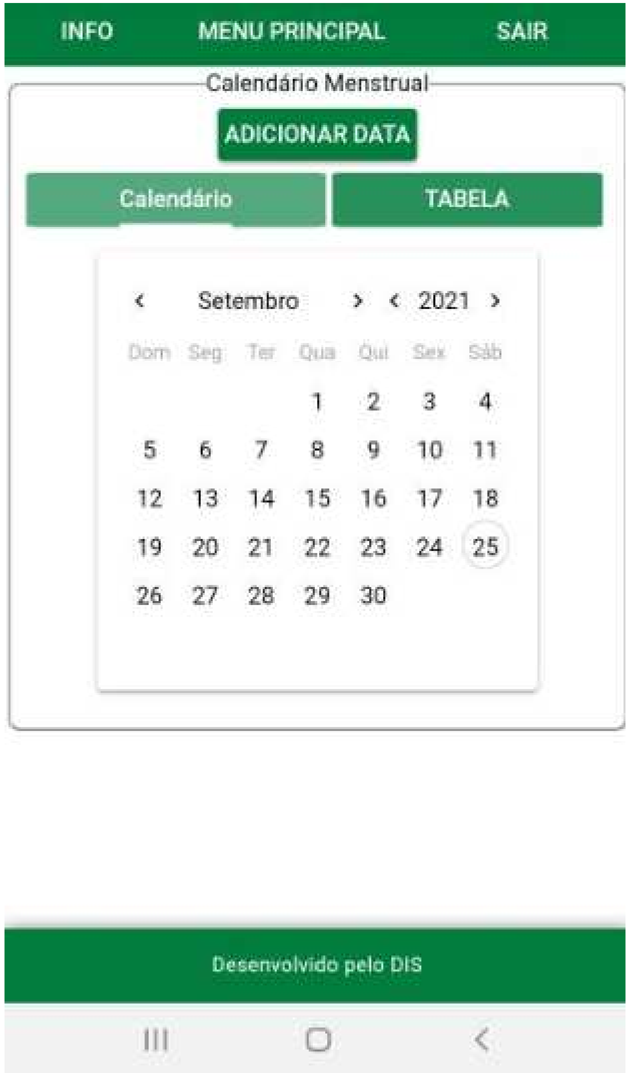

- ✓ **Este é o menu do CALENDÁRIO MENSTRUAL**
- ✓ **Você poderá anotar a data de sua última menstruação e também visualizar todas as outras datas registradas**

PACIENTE

CALENDÁRIO MENSTRUAL – ADICIONAR DATA

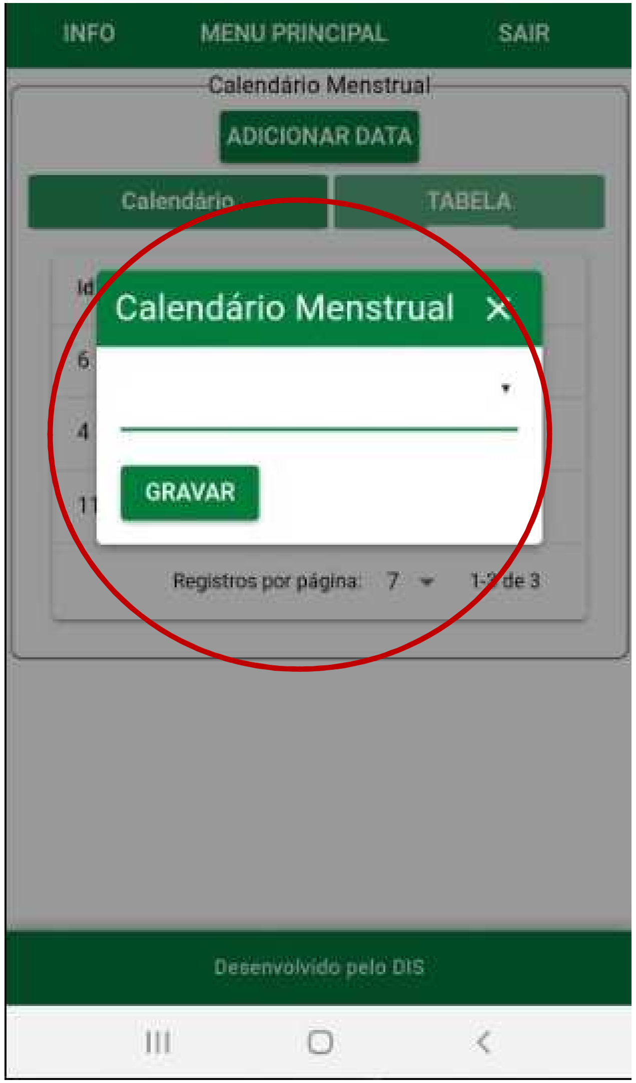

- ✓ Ao apertar em ADICIONAR DATA, aparecerá essa janela para anotar a data de sua última menstruação
- ✓ Depois clique em GRAVAR

PACIENTE

CALENDÁRIO MENSTRUAL - TABELA

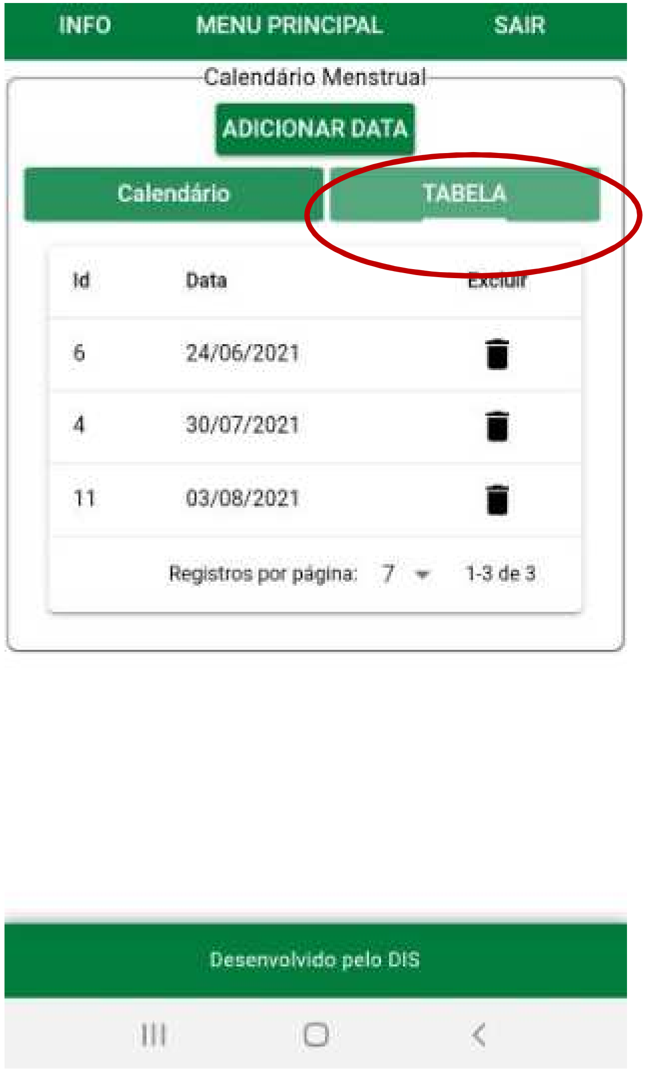

- ✓ Ao apertar em TABELA, aparecerão todas as data de sua última menstruação que foram anotadas por você ou pelo eu médico
- ✓ Depois clique em GRAVAR

Universidade Federal de São Paulo

Hospital Universitário / Hospital São Paulo

Ambulatório de Doença Trofoblástica

Gestacional

Logotipo utilizado no aplicativo
